# Prediction of backbone dihedral angles and protein secondary structure using support vector machines

**DOI:** 10.1186/1471-2105-10-437

**Published:** 2009-12-22

**Authors:** Petros Kountouris, Jonathan D Hirst

**Affiliations:** 1 School of Chemistry, University of Nottingham, University Park, Nottingham NG7 2RD, UK

## Abstract

**Background:**

The prediction of the secondary structure of a protein is a critical step in the prediction of its tertiary structure and, potentially, its function. Moreover, the backbone dihedral angles, highly correlated with secondary structures, provide crucial information about the local three-dimensional structure.

**Results:**

We predict independently both the secondary structure and the backbone dihedral angles and combine the results in a loop to enhance each prediction reciprocally. Support vector machines, a state-of-the-art supervised classification technique, achieve secondary structure predictive accuracy of 80% on a non-redundant set of 513 proteins, significantly higher than other methods on the same dataset. The dihedral angle space is divided into a number of regions using two unsupervised clustering techniques in order to predict the region in which a new residue belongs. The performance of our method is comparable to, and in some cases more accurate than, other multi-class dihedral prediction methods.

**Conclusions:**

We have created an accurate predictor of backbone dihedral angles and secondary structure. Our method, called DISSPred, is available online at http://comp.chem.nottingham.ac.uk/disspred/.

## Background

The rapid growth of the number of protein sequences has far outpaced the experimental determination of their structures, but knowledge of the three dimensional structure of a protein can help to determine its function. Thus, computational methods are often used to predict the structures of sequences for which no experimental information is available. Such approaches are based on the premise that all the information needed to determine the three dimensional structure is encoded in the amino acid sequence [1]. A critical first step is the accurate prediction of the protein secondary structure, the local, regular structure defined by hydrogen bonds. Over the past 40 years, researchers have been predicting secondary structure with various approaches. Notably, the predictive accuracy has improved substantially over the past 20 years through the use of evolutionary information and machine learning algorithms [2]. In 1988, Qian and Sejnowski pioneered the application of artificial neural networks (ANNs) to predict secondary structure [3]. Different ANN architectures have been used to predict the secondary structure, such as feed-forward back-propagation ANN [4-6], bidirectional recurrent ANN [7], cascade-correlation ANN [8] and cascaded ANN with linear discriminant analysis [9]. The most successful methods in the 1990s, such as PHD [4] and PSIPRED [6], used multi-layer feed-forward ANNs and achieved predictive accuracies of around 77%-78%. Moreover, other approaches have been used over the past 20 years, such as analysis with hidden Markov models [10,11], multiple linear regression [12,13] and, more recently, non-linear dynamic systems [14]. Other predictors, such as JPRED [15,16], make consensus secondary structure predictions. Since 2001 [17], the support vector machine method (SVM) has been applied to predict secondary structure [18-21]. PMSVM [18] enhanced the prediction of the single SVM scheme with a dual-layer SVM approach. More recently, YASSPP [21] improved the SVM-based predictions by combining position-specific and nonposition-specific information with better kernel functions. Despite relatively accurate predictions, there is still an opportunity for additional information and novel methods to boost the predictions.

The backbone dihedral angles, *ϕ *and *ψ*, can provide important information about the three dimensional structure of the protein. They vary from -180° to +180°, but they cannot adopt all possible values, because of steric restrictions. The famous Ramachandran plot [22] illustrates the sterically allowed regions of the dihedral angles. The experimental determination of dihedral angles is usually time-consuming and expensive, but can be accelerated by algorithms that use sequence information and chemical shifts [23]. Accurate prediction of dihedral angles can facilitate tertiary structure prediction. It has been suggested that if none of the dihedral angles of an eight-residue fragment differs from another eight-residue fragment by more than 120°, the RMSD between the two segments is less than 1.4Å [24]. The Rosetta server [25], the most successful method for three dimensional structure prediction, uses predictions from HMMSTR [26] of the secondary structure and the dihedral angles, which are described with an alphabet of eleven states. Apart from protein structure modelling, predicted dihedral angles have been used successfully to improve sequence alignment [27], fold recognition [28] and secondary structure prediction [8,29]. Early studies used simple models to explore protein conformational space and facilitate 3D structure prediction [30,31]. Over the past few years, several methods have been developed to predict dihedral angles based on different numbers of structural states. De Brevern and colleagues [32] used self-organising maps and hidden Markov models to identify a structural alphabet of 16 "protein blocks". This alphabet was used in LOCUSTRA [33] and by Dong and colleagues [34] to predict structural states using SVMs and ANNs, respectively. Kuang and colleagues [35] developed an SVM-based method that makes three-state and four-state predictions with an accuracy of 78.7% and 77.3%, respectively, based on the dihedral regions proposed by a previous study [36]. DHPRED [37], another SVM-based method, achieved 80% three-state accuracy based the dihedral regions defined by Lovell and colleagues [38]. The definition of the dihedral angle regions is important in this kind of approach. Other methods predict the real value of the dihedral angles. ANGLOR [39] uses ANNs and SVMs to predict the backbone ϕ and ψ angle, respectively. Furthermore, Zhou and co-workers developed Real-SPINE [40-42], a method that predicts the real-valued dihedral angles, using consensus predictions from five ANNs. Real-SPINE has achieved the best mean absolute error [42] and correlation coefficient [41] reported to date.

The backbone dihedral angles and the secondary structure elements are highly correlated and, therefore, can be used together to boost the predictions. DESTRUCT [8] implemented this idea using an iterative set of cascade-correlation neural networks to predict independently both the real value *ψ *angle and the secondary structure and it used the results to enhance the predictions. The predictive secondary structure accuracy on a non-redundant set of 513 proteins [43] is, until now, the highest reported score on that particular dataset. Even though the dihedral prediction was limited, it provided additional information, which improved the secondary structure prediction significantly. Furthermore, the inclusion of secondary structure prediction improved the *ψ *angle prediction.

Here, we take the approach one step forward. Using various definitions of dihedral states created by two unsupervised machine learning algorithms, our method improves the predictions of backbone dihedral angles and secondary structure. Multi-state dihedral prediction offers some advantages over real-value prediction, such as easy sampling and detailed analysis of the dihedral space. Moreover, clustering techniques, often called class discovery techniques, can provide important insight into specific regions of the dihedral space which cannot be easily addressed with real-value prediction. We use the SVM method, which is superior in many practical applications, because it finds the optimal hyperplane to separate two classes. The results we present in this paper show that our method predicts the three-state secondary structure significantly more accurately than other contemporary methods, due to the dihedral information used. Additionally, the multi-state predictive accuracy of dihedral clusters enhanced with predicted secondary structures is comparable to, and in some cases more accurate than, other methods.

## Methods

### Support Vector Machines

The SVM [44] is an algorithm for learning classification and regression rules from data. The SVM method has become an area of intense research, because it performs well with real-world problems, it is simple to understand and implement and, most importantly, it finds the global solution, while other methods, like ANNs, have several local solutions [45]. The SVM can find non-linear boundaries between two classes by using a kernel function, which maps the data from the input space into a richer feature space, where linear boundaries can be implemented. Furthermore, the SVM effectively handles large feature spaces, since it does not suffer from the "curse of dimensionality", and, therefore, avoids overfitting, a common drawback of supervised learning techniques.

Since an SVM is a binary classifier, it cannot be used to separate data with more than two classes. However, several techniques allow combinations of SVM models to create a multi-class SVM method. The most popular methods are called *one-against-one *and *one-against-all*. The former constructs  binary models for *n *classes and each one trains on data from two different classes. A voting scheme is applied at the end to decide the final prediction. The one-against-all method constructs *n *binary models for *n *classes and each one decides whether an instance belongs to a class or not. At the end, winner-takes-all decides the final prediction. In this work, we use the LibSVM package [46], which offers multi-class SVM using the one-against-one approach.

The ultimate goal is to classify previously unseen examples correctly. Therefore, it is not useful to achieve high training accuracy if the prediction is not accurate enough. In order to estimate the generalisation error, we use *n*-fold cross-validation. The training data are split into *n *subsets and, sequentially, *n *- 1 of them are used for training and the remaining one for testing. This approach is repeated *n *times, until all subsets are used once for testing. In our case, 10-fold cross-validation was used.

The selection of the kernel function, which maps the input data into a high-dimensional feature space, is one of the main challenges in SVM methods. The radial basis function (RBF), shown in equation 1, is the most suitable kernel function for complex problems. Secondary structure prediction appears to be such a problem and RBF has been used by the majority of SVM-based secondary structure prediction methods [17-19]. Hence, we use the RBF kernel.(1)

where **x**_i _and **x**_j _are the input vectors for instances *i *and *j*, respectively. To optimise the learning process, one can adjust parameters *C *and *γ*. The regularisation parameter, *C*, controls the trade-off between training error and the margin that separates the two classes, while *γ *controls the width of the RBF kernel. The parameter optimisation was performed using a grid search approach, where pairs of (*C*, *γ*) were tried on the training set and the one with the best cross-validated accuracy was selected. A practical method [47] to identify good parameters is to try exponentially growing sequences of *C *and *γ*. We tried the following values: *C *= 2^-5^, 2^-3^, ..., 2^15 ^and *γ *= 2^-15^, 2^-13^, ..., 2^3^. After the best pair of values was found, a finer search on that specific region was conducted to identify the optimal values. Here, the optimised parameters for CB513 dataset were found to be *C *= 1.5 and *γ *= 0.08. However, the predictive accuracy was similar for *C *and *γ *in the ranges [1,2] and [0.06, 0.1], respectively.

### Clustering of dihedral angles

There is no clear optimal way to separate the dihedral space into regions in order to provide structural information. Other dihedral prediction methods [33,35,37] have used various definitions of the dihedral angle regions, taken from previous studies [32,36,38]. Here, we attempt to discover the best clusters using two unsupervised machine learning techniques, k-Means and expectation maximisation (EM), that group a given collection of patterns into clusters based on a similarity measure [48]. This approach is often called data clustering and has been successfully used in bioinformatics, especially to identify new classes for gene expression data [49]. Both the clustering algorithms we use are partitional methods, which divide the data into *k *clusters without overlap and each cluster can be represented by a centroid.

*K*-*Means *[50] is one of the simplest and fastest clustering algorithms. The main goal is to divide a dataset into *k *clusters, where *k *must be defined a priori. It starts with an initial selection of *k *centroids, which is usually random, and keeps reassigning the data points into clusters based on the similarity between the data point and the cluster centres, until a convergence criterion is met. Euclidean distance is used as a similarity measure in our method. The k-Means algorithm is popular, because it is easy to implement, and its time complexity is *O*(*n*), where *n *is the number of instances in the dataset. A drawback is that the algorithm is sensitive to the selection of the initial partition and may converge to a local minimum [48].

Another way to tackle clustering problems is using Gaussian mixture models, in which the underlying assumption is that the data points are drawn from one of *k *Gaussian distributions with mean *μ*_*i *_and standard deviation *σ*_*i*_. The goal is to identify the parameters of each Gaussian distribution. The most popular algorithm in this case is the *Expectation Maximisation *(EM) algorithm [51], whose steps are similar to those of the k-Means algorithm. EM starts with a random assignment of the *k *Gaussian distribution parameters, *μ*_*i *_and *σ*_*i*_, and computes the cluster probability for each data point based on the probability density function. The probability parameters are re-estimated and the procedure is repeated until a termination criterion is met. EM is useful when one wants to identify and separate several probability distributions in the data. On the other hand, like k-Means, EM can get stuck in local minima [48].

We used the WEKA implementations [52] of the above algorithms to cluster the dihedral space into regions. In order to study many different partitions, we used different numbers of clusters, from two to 12. To prevent the algorithms from getting stuck in local minima, the clustering process was carried out several times with different initial partitions. Because of periodicity, the dihedral angles +180° and -180° are identical. However, this cannot be captured by distance-based clustering algorithms, like k-Means and EM. In order to reduce the effect of the angle periodicity, we perform a single transformation of the *ψ *angle, originally proposed [41] in Real-SPINE 2.0, by shifting the *ψ *angles between -180° and -100° by 360°. Hence, the *ψ *angles were in the range -100° and +260°. There are few dihedral angles with values at either end of this range, which improves the clustering.

### Datasets and SVM design

DISSPred was trained and tested on three different datasets. The first was CB513 [43], a non-redundant non-homologous set of 513 protein sequences. CB513 was used to study the impact of various input coding schemes and to tune the kernel parameters. All 513 proteins have less than 25% sequence similarity to ensure that there is very little homology in the training set. Since CB513 was used to train many secondary structure prediction methods, we can compare the cross-validated accuracy of our method directly with other methods. The second dataset was PDB-Select25 (version October 2008) [53], a set of 4018 chains from the PDB with less than 25% sequence similarity and X-ray resolution less than 3.0 Å. After removing chains with regions of unknown structure, the final dataset contained 3978 chains from 3852 proteins with a total number of 560 073 residues. In order to make the training process faster and validate the performance on an independent dataset, PDB-Select25 was divided into two subsets, one of which was used for training and the other one for testing. The subsets have approximately the same composition of three-state secondary structure elements: 35% for helix, 23% for strand and 42% for coil. Moreover, we ensured that both datasets have a similar distribution of small/large protein chains. Thus, subset one contains 280 128 residues from 1989 chains, whereas subset two contains 279 945 residues from 1988 chains. Finally, we also report DISSPred's predictive accuracy on four subsets of the dataset provided by the EVA secondary structure prediction server [54]. The PDB codes and chain identifiers as well as the SCOP class [55] of each chain in the above datasets are listed at DISSPred's website http://comp.chem.nottingham.ac.uk/disspred.

The secondary structure can be assigned using DSSP [56], STRIDE [57] or DEFINE [58]. Here, we use DSSP, the most established method, which assigns the secondary structure using eight states: H (*α*-helix), G (3_10_-helix), I (π-helix), E (extended *β*-strand), B (isolated *β*-bridge), T (turn), S (bend) and "_" (other/coil). Most of the existing methods predict the secondary structure using a three-state assignment. Therefore, we reduce the above representation to three states, by assigning H, G and I to the helix state (H), E and B to the extended state (E) and the rest (T, S and "_") to the coil state (C).

Since their first use by PSIPRED [6], PSI-BLAST [59] position specific scoring matrices (PSSMs) are employed by the majority of secondary structure prediction methods. PSSMs are constructed using multiple sequence alignments and they provide crucial evolutionary information about the structure of the protein. PSSMs have *N *× 20 elements, where the *N *rows correspond to the length of the amino acid sequence and the columns correspond to the 20 standard amino acids. PSSMs represent the log-likelihood of a particular residue substitution, usually based on a weighted average of BLOSUM62 [60], and are created using the PSI-BLAST algorithm. We generated the PSSMs using the BLOSUM62 substitution matrix with an E-value of 0.001 and three iterations against the nr database, which was downloaded in February 2009. The data were filtered by *pfilt *[61] to remove low complexity regions, transmembrane spans and coiled coil regions. The PSSM values were linearly scaled simply by dividing them by ten. Typically, PSSM values are in the range [-7,7] but some values outside this range may appear. Linear scaling maintains the same distribution in the input data and helps avoid numerical difficulties during training.

We used different coding schemes for the secondary structure prediction and the dihedral angle prediction. After testing different local window sizes (*w*), we selected *w *= 15 for secondary structure prediction and *w *= 11 for dihedral prediction, which give the highest predictive accuracy for each case. Hence, using the PSSM values for each residue, the input vector has length 15 × 20 for secondary structure prediction and 11 × 20 for prediction of dihedral angles. A local window, rather than just the individual residue, allows the classifier to capture useful additional information [2].

**Figure 1:**
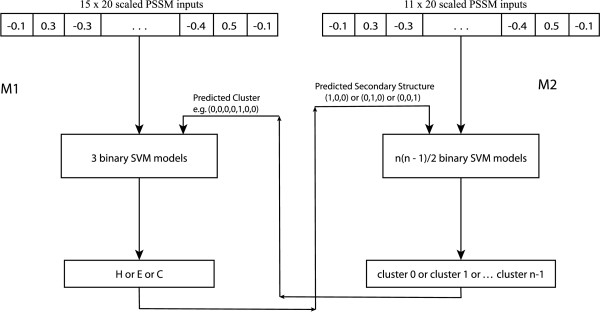
**Schematic representation of our method**. Firstly, the PSSM-only predictions are calculated. Then, they are used to augment the input vector and enhance the results.

Our method consists of two different models, *M*1 and *M*2, that predict secondary structure and backbone dihedral angles, respectively (figure 1). *M*1 uses a local window of 15 residues. The input vector of *M*1 contains 15 × 20 scaled PSSM values, 20 for each residue in the fragment, and the output is one of the three states of secondary structure: H, E or C. *M*2 uses a shorter window of 11 residues and the input vector consists of 11 × 20 scaled PSSM values. The output of the model is an integer in the range [0, *n *- 1], where *n *is the number of clusters used to identify the dihedral angle regions. We systematically partitioned the dihedral space into different numbers of clusters, from two to 12. After the first run of the models using only the PSSM values, the input vector of *M*1 was augmented with *n *binary values, which were equal to unity if the residue was predicted to be in that particular cluster and zero otherwise. Only one of the *n *values can be equal to unity, since the residue is predicted into a single cluster. Similarly, the input vector of *M*2 was augmented with three binary values, one for each secondary structure. This second stage is iterated several times to improve the predictions further. In other words, the predicted secondary structures from model *M*1 and the predicted dihedral clusters from model *M*2 at step *m *are used to augment the input vector of models *M*2 and *M*1 respectively at step *m *+ 1.

### Prediction accuracy assessment

We used several measures to assess the performance of DISSPred, most of them defined in the EVA server [54]. Q_3 _is the three-state overall percentage of correctly predicted residues:(2)

where *N*_*res *_is the total number of residues and *M*_*ij *_is the number of residues observed in state *i *and predicted in state *j*, with *i *and *j *∈ {H, E, C} (i.e. *M*_*ii *_is the number of residues predicted correctly in state *i*). In the case of dihedral prediction, *i *and *j *can be any number from 0 to *nc *- 1, where *nc *is the number of clusters. Moreover, we calculate the per-state accuracy, the percentage of correctly predicted residues in a particular state:(3)

where *obs*^*i *^is the number of residues observed in state *i*. Additionally, the Matthew's correlation coefficient [62], *C*_*i*_, provides a measure for the performance at each state:(4)

Finally, ErrSig is the significant deviation for three-state accuracy, a measure used to distinguish between two methods. It is defined as the standard deviation divided by the square root of the number of proteins (SD/).

We use two additional measures to assess the accuracy of dihedral prediction. Firstly, the mean absolute error (MAE) is the average of the absolute distance between the predicted and the real (observed) value, *p *and *x*, respectively. In order to take in account the periodicity of the dihedral angles, the MAE is calculated by:(5)

The predicted value corresponds to the centre of the predicted cluster. Finally, it is interesting to see the fraction of residues whose dihedral angles are predicted close to the real value. Q_30 _score is the percentage of residues whose predicted value is within 30° of the real value.

## Results and Discussion

In the additional file 1, the cluster centroids and the standard deviation of each cluster are shown, while additional file 2 shows all the different partitions of the *ϕ *- *ψ *space as well as the distribution of secondary structure element in each cluster. The helical residues belong mainly to one compact, highly-populated cluster, while there are clusters that consist mostly of strand residues, the most difficult secondary structure element to predict. For the above reason, the predictive accuracy of both helical and extended residues is improved significantly after the predicted dihedral information is used. On the other hand, the coil residues are distributed in different clusters, which makes their prediction more difficult.

**Figure 2:**
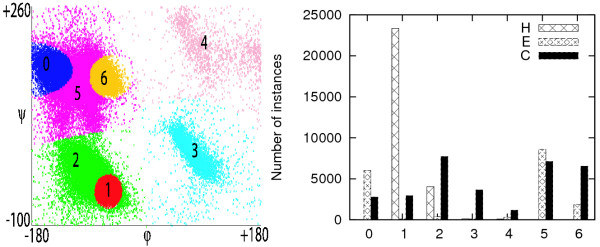
**Clustering of the dihedral angles using EM clustering with seven clusters (left) and the distribution of secondary structure in every cluster (right)**.

Table 1 shows the predictive accuracy of the secondary structure at every stage of the iterative algorithm. Our method achieves a cross-validated predictive accuracy of 80% after the first iteration when using predicted dihedral data from EM clustering with seven clusters (figure 2). There is an improvement of 1.7% in the predictive accuracy when the predicted dihedral clusters are used together with the PSSM values. The Q_3 _score does not improve in the subsequent iterations of the method. However, the predictive accuracy of helical and extended residues in some cases improves after each iteration; it is up to 3.6% and 3% higher, respectively, after the third iteration. On the other hand, the prediction of coil residues decreases slightly for a small number of clusters, but it increases for large number of clusters after the first iteration. In general, the prediction of coil residues is not improved significantly when the dihedral information is used. The explanation can be derived from the Ramachandran plot. The coil residues are not highly localised in *ϕ *- *ψ *space and, since there are no compact coil clusters, the dihedral information given to the classifier is not particularly useful. Interestingly, regardless of the clustering algorithm or the number of clusters used, the predictive accuracy improves significantly after the first iteration, showing that even limited dihedral information can boost the secondary structure prediction. Finally, the application of the smoothing rules originally proposed in PHD [4], which were used to improve the performance of DESTRUCT [8], did not improve the predictive accuracy of DISSPred.

**Table 1:** The secondary structure prediction for CB513 dataset after three iterations.

CB513													
		**DHR-1st run**				**DHR-2nd run**				**DHR-3rd run**			
		
**Category**	**NC**	**Q_3 _(%)**	**Q_*H *_(%)**	**Q_*E *_(%)**	**Q_*C *_(%)**	**Q_3 _(%)**	**Q_*H *_(%)**	**Q_*E *_(%)**	**Q_*C *_(%)**	**Q_3 _(%)**	**Q_*H *_(%)**	**Q_*E *_(%)**	**Q_*C *_(%)**

PSSM-only	0	78.3	80.3	66.7	82.6								

EM	2	79.3	82.3	68.5	82.5	78.3	81.1	68.0	81.2	79.0	83.0	69.1	81.0
	3	79.4	82.6	68.7	82.3	78.3	81.3	68.0	81.0	79.1	83.8	68.9	80.6
	4	79.5	82.8	68.6	82.4	78.3	81.3	68.0	81.0	79.2	83.8	69.0	80.8
	5	79.9	83.4	69.2	82.7	78.4	81.2	68.2	81.1	79.5	83.6	69.3	81.4
	6	80.0	83.7	68.9	82.8	78.2	81.2	68.2	80.8	79.5	83.6	69.3	81.3
	7	**80.0**	**83.3**	**69.0**	**83.1**	78.2	81.4	67.9	80.9	79.4	83.6	69.3	81.3
	8	79.8	82.9	68.6	83.1	78.1	81.7	67.8	80.2	79.4	84.2	69.3	81.6
	9	79.9	82.8	69.2	83.2	78.2	81.6	67.9	80.7	79.3	84.3	69.5	80.3
	10	79.8	83.0	68.5	83.3	78.3	81.7	68.2	80.6	79.4	83.7	69.7	80.9
	11	79.6	82.4	68.5	83.2	78.2	81.6	67.9	80.5	79.5	83.6	69.6	81.4
	12	79.9	82.7	68.7	83.5	78.2	81.8	67.7	80.3	79.5	83.6	69.5	81.4

k-Means	2	79.3	82.1	68.8	82.5	78.2	81.1	68.2	81.1	74.7	84.4	62.2	73.1
	3	79.6	82.8	69.2	82.4	78.2	81.3	68.0	81.1	74.8	85.0	61.8	72.9
	4	79.9	83.4	68.8	82.7	78.2	81.4	67.9	81.0	79.3	83.7	68.6	81.3
	5	79.9	83.3	69.1	82.7	78.2	81.5	67.8	80.9	79.2	83.9	68.8	80.7
	6	79.9	83.4	68.6	82.9	78.1	81.5	67.8	80.8	79.0	83.5	68.8	80.6
	7	79.9	83.3	67.9	83.3	78.0	81.6	67.7	80.5	79.2	83.7	68.8	80.8
	8	79.7	82.9	68.3	83.1	78.0	81.6	67.5	80.5	79.3	83.8	68.8	80.9
	9	79.8	83.4	67.7	83.2	78.0	81.7	67.4	80.5	79.3	83.6	68.4	81.4
	10	79.7	82.8	67.7	83.4	78.0	81.7	67.5	80.5	79.3	83.5	68.3	81.6
	11	79.8	83.0	69.0	82.9	78.0	81.6	67.6	80.4	79.2	83.9	68.9	80.7
	12	79.7	83.2	68.1	83.1	78.1	81.0	68.0	81.1	79.2	82.9	68.7	81.5

The accuracy from the initial PSSM-only prediction is shown in the first row. In bold are the most accurate predictions based on Q_3_. NC = number of clusters used to predict dihedral angles, DHR = input vector augmented by predicted dihedral cluster,

Table 2 shows a comparison of DISSPred with other secondary structure prediction methods. Most of the measures presented show that DISSPred is more accurate than other predictors. The three state accuracy (Q_3_) achieved is over 2% higher than other SVM-based methods (YASSPP [21], PMSVM [18], SVMfreq [17] and SVMpsi [19]). Moreover, the predictive accuracy is higher than the accuracy reported by the most successful methods that use multi-layer ANNs (PSIPRED [6] and PHD [4]). The difference is larger than the value of ErrSig measure (0.5), which shows that DISSPred is significantly more accurate than other methods. DESTRUCT [8], which achieves the closest Q_3 _accuracy to our method, also uses predicted dihedral information to boost the results, which highlights the utility of predicted dihedral angles in secondary structure prediction.

**Table 2:** Comparison of cross-validated predictive accuracy on CB513 dataset with other secondary structure methods.

Method	Q_3 _(%)	Q_*H *_(%)	Q_*E *_(%)	Q_*C *_(%)	C_*H*_	C_*E*_	C_*C*_
DISSPred	80.0 ± 0.5	83.3	69.0	83.1	0.77	0.68	0.62
PSIPRED	78.2	N/A	N/A	N/A	N/A	N/A	N/A
PHD	74.7	N/A	N/A	N/A	N/A	N/A	N/A
DESTRUCT	79.4	N/A	N/A	N/A	N/A	N/A	N/A
YASSPP	77.8	N/A	N/A	N/A	0.71	0.64	0.58
PMSVM	75.2	80.4	71.5	72.8	0.71	0.61	0.61
SVMfreq	73.5	75.0	60.0	79.0	0.65	0.53	0.54
SVMpsi	76.6	78.1	65.6	81.1	0.68	0.60	0.56

The results for PSIPRED, PHD and DESTRUCT were obtained from reference [8].

Table 3 shows the analysis of the results for the two main types of secondary structure: helix and strand. In particular, we analyse the predictions for helices and sheets with more than three and more than two residues, respectively. We assume that a secondary structure element is correctly predicted if more than 65% of its residues are predicted correctly. We find that 83.7% of the helices and 72.6% of the strands are predicted correctly. Furthermore, we divide the secondary structure elements into three categories based on their length. Interestingly, long strands are more difficult to predict than the short ones, whereas long helices are predicted more accurately than the short ones. Finally, the terminal residues of the secondary structure elements are more difficult to predict, with the N-terminal residues predicted better than the C-terminal residues, particularly in helices.

**Table 3:** Prediction of the two main types of secondary structure: helix and strand.

CB513		
**Measure**	**Helix (*l *≥ 4 res)**	**Stand (*l *≥ 3 res)**

Q_>65_	83.7%	72.6%
Short (*l* ≤ 8)	65.6%	74.8%
Med (8 <*l* ≤ 15)	94.4%	57.1%
Long (*l* > 15)	97.3%	27.3%
N-term res	73.4%	62.7%
C-term res	62.5%	59.1%

*l*: length of the secondary structure element

Q_>65_: the percentage of elements that have more than 65% of their residues predicted correctly

Short: Q_>65 _of elements with length up to eight residues

Med: Q_>65 _of elements with length between nine and 15 residues

Long: Q_>65 _of elements with length more than 15 residues

N-term res: the percentage of elements whose first residue (N-terminal) is predicted correctly

C-term res: the percentage of elements whose last residue (C-terminal) is predicted correctly.

It is interesting to analyse how the predictive ability changes in every cluster when the predicted dihedral angles are used, shown in additional file 3. Unsurprisingly, the prediction accuracy improves the most in clusters that contain mainly helical residues. In particular, the clusters with centroids around (-62°, -40°), which mainly consist of residues in right-handed helices, and the clusters with centroids around (75°, 17°), which mainly consist of residues in left-handed helices, show significant improvement. Moreover, clusters that contain mainly strand residues are also predicted more accurately. On the other hand, clusters that contain mainly coil residues or mixed strand/coil or helix/coil residues do not show any significant improvement. In fact, in some cases the additional dihedral information can decrease the predictive accuracy. However, these clusters are not highly populated and, therefore, do not affect the overall accuracy significantly.

Table 4 shows the predictive accuracy of dihedral angle regions, using different number of clusters (from two to 12), with two different clustering algorithms. The predictive accuracy improves significantly after the second run of the method, mainly due to the improved secondary structure prediction (see Table 1). Although the EM algorithm performs worse than the k-Means algorithm for two and three clusters, it gives more accurate results for the rest of the partitions. Interestingly, our method performs particularly well for a small number of clusters; it achieves predictive accuracy over 80% for two, three and four regions of dihedral space. It outperforms other multi-class prediction methods. Kuang et al. [35] reported three-state accuracy of 78.7% and four-state accuracy of 77%. The HMMSTR [26] alphabet can be transformed into four states with a predictive accuracy of 74% [35]. Moreover, DHPRED [37] achieved three-state accuracy of around 81% while LOCUSTRA [33] reports three-state accuracy of 79.2%. DISSPred achieves a three-state accuracy of 81.2% and a four state accuracy of 80.5%, using the EM clustering algorithm.

**Table 4:** The cross-validated accuracy of dihedral prediction on CB513 dataset.

CB513								
	**PSSM-only**		**SSE-1st run**		**SSE-2nd run**		**SSE-3rd run**	
	
**NC**	**EM (%)**	**k-Means (%)**	**EM (%)**	**k-Means (%)**	**EM (%)**	**k-Means (%)**	**EM (%)**	**k-Means (%)**

2	81.4	81.7	81.8	82.1	83.2	83.4	81.8	83.5
3	79.3	79.6	79.7	79.8	81.2	81.1	79.6	81.2
4	78.7	74.5	79.0	74.4	80.5	76.1	79.0	75.8
5	65.0	63.8	65.2	64.1	66.9	65.3	65.2	65.0
6	63.7	59.2	63.8	59.3	65.5	60.4	63.7	60.1
7	56.5	54.6	56.8	54.7	58.3	56.0	56.8	55.4
8	53.8	53.7	54.0	53.8	55.4	55.1	53.9	54.6
9	53.8	51.1	54.0	51.0	55.3	52.3	54.0	51.7
10	52.9	50.2	53.1	50.3	54.5	51.6	53.0	51.0
11	50.3	48.5	50.6	48.5	51.8	49.7	50.6	49.1
12	47.0	41.2	47.2	41.5	48.4	42.3	47.2	42.1

NC: the number of clusters,

PSSM-only: input vector with only PSSM values,

SSE: input vector augmented with predicted secondary structure elements.

**Figure 3:**
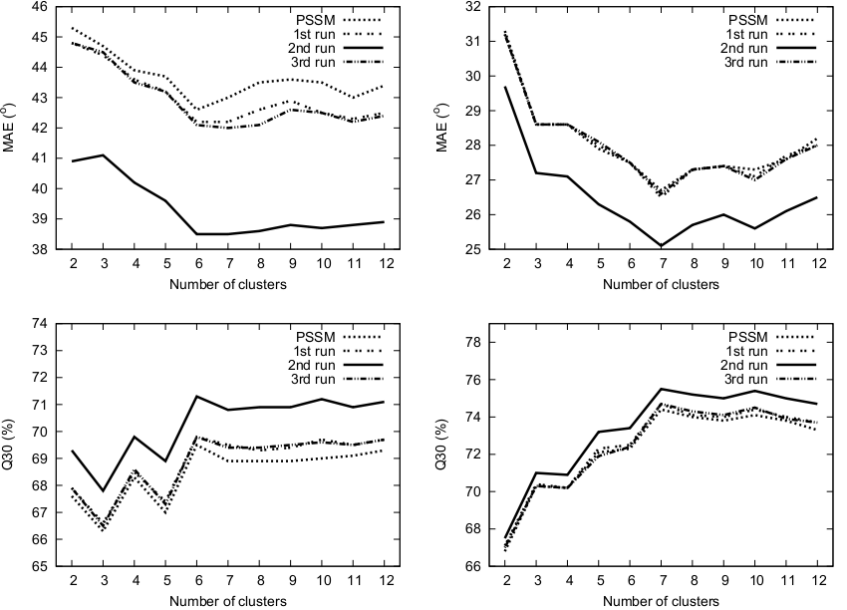
**Top: the mean absolute error (MAE) after each iteration of the method for *ψ *angles (left) and *ϕ *angles (right)**. Bottom: the percentage of predicted dihedral angles within 30° (Q_30_) of the real values for *ψ *angles (left) and *ϕ *angles (right).

On the other hand, although the predictive accuracy is low for large number of clusters, the predictions can provide important information about the local structure. We explore this by calculating the MAE and Q_30 _score. Figure 3 shows that the MAE decreases and Q_30 _increases as we increase the number of clusters after each iteration using EM clustering. The best results are obtained after the second iteration, which is in agreement with the predictive accuracy shown in table 4. Additional file 4 shows the results for the MAE and Q_30 _score using all different numbers of clusters with EM clustering after the second iteration. Six and seven clusters give the lowest MAE and the highest Q_30 _score and are presented in table 5. Therefore, the structural information contained in a dihedral prediction does not necessarily depend on the predictive accuracy. In fact, the improvement of secondary structure prediction was higher when we used predicted dihedral data from six and seven clusters. Notably, the MAE of our method is comparable to the MAE reported by Real-SPINE 2.0 and 3.0 [41,42], even though we only predict dihedral states instead of real value dihedral angles. Real-SPINE 3.0 [42] has MAEs of 36° for the *ψ *angle (20°, 32° and 56° for helix, strand and coil, respectively) and 22° for the *ϕ *angle (10°, 25° and 34° for helix, strand and coil, respectively). Moreover, LOCUSTRA [33] reports MAEs of 24.7° and 38.4° for *ϕ *and *ψ*, respectively, while ANGLOR [39] achieves MAEs of 28° and 46° for *ϕ *and *ψ*, respectively. Since, the above methods are trained on different datasets, their MAEs should not be compared directly. We present them here just to give a rough comparison between the methods.

**Table 5:** The MAE and Q_30 _using six and seven clusters with EM clustering.

CB513				
	*ϕ ***angle**		*ψ ***angle**	
		
**No. clusters**	**6**	**7**	**6**	**7**

MAE (°)	25.8	25.1	38.5	38.5
MAE_*H *_(°)	12.2	11.3	22.3	19.7
MAE_*E *_(°)	24.1	25.4	30.7	33.7
MAE_*C *_(°)	38.4	36.9	56.7	57.3
Q_30 _(%)	73.4	75.6	71.3	70.8
QH_30 _(%)	90.4	91.9	88.0	89.5
QE_30 _(%)	72.9	72.2	78.3	76.2
QC_30 _(%)	59.0	63.3	53.0	51.8

The mean absolute error (MAE) and the percentage of predicted dihedral angles within 30° of the real value (Q_30_) for both backbone dihedral angles *ϕ *and *ψ *after two iterations, using six and seven clusters with EM clustering.

Tables 6 and 7 show the MAE for *ϕ *and *ψ*, respectively, for each amino acid. We use the number of clusters that gives the lowest overall MAE (Table 5), which are seven clusters for *ϕ *and six clusters for *ψ*. Glycine has the largest error for both angles, because is the smallest and the most flexible amino acid and can take many different conformations without steric restrictions. On the other hand, proline has the smallest MAE for *ϕ*, because its ring structure restricts the *ϕ *angle to around -60°. Amino acids that have strong helical preferences [63], such as alanine, methionine and glutamic acid, have lower MAEs than the others. On the other hand, amino acids with a high hydropathy index [64], such as leucine, isoleucine and valine, also have low MAEs. These residues are usually densely packed in the hydrophobic protein core and, hence, they have limited flexibility compared to residues on the hydrophilic surface. Finally, apart from glycine and proline, residues that have coil preferences, such as asparagine and serine, have the highest MAEs.

**Table 6:** The MAE of each amino acid for *ϕ *angle.

CB513 *ϕ*-angle				
**AA**	**MAE (**°**)**	**MAE_*H*_(°)**	**MAE_*E*_(°)**	**MAE_*C*_(°)**

A	21.0	9.0	29.2	36.7
R	23.0	9.6	25.2	38.8
N	37.4	16.0	35.4	48.6
D	29.1	11.3	32.6	38.6
C	25.8	14.1	21.5	37.9
Q	22.1	9.4	28.1	36.7
E	21.2	9.1	27.2	36.7
G	60.3	32.4	86.9	64.8
H	30.9	15.9	31.7	41.6
I	17.0	9.7	16.4	29.0
L	17.8	9.1	19.2	31.7
K	23.3	10.3	27.0	35.6
M	19.7	9.8	23.9	32.4
F	24.2	12.5	22.3	39.3
P	13.4	10.4	13.1	14.2
S	30.1	13.2	34.8	38.6
T	24.1	12.7	22.2	32.4
W	24.5	12.6	27.3	36.5
Y	25.3	12.5	24.9	40.2
V	18.0	9.6	17.0	30.1

The mean absolute error (MAE) of each amino acid for *ϕ *angle after two iterations, using seven clusters with EM clustering.

**Table 7:** The MAE of each amino acid for *ψ *angle.

CB513 - *ψ *angle				
**AA**	**MAE(°)**	**MAE_*H*_(°)**	**MAE_*E*_(°)**	**MAE_*C*_(°)**

A	32.9	18.1	32.8	57.9
R	35.3	17.5	33.4	59.2
N	45.5	23.4	46.3	56.7
D	44.8	23.6	43.0	57.9
C	42.9	37.0	29.1	58.2
Q	35.0	16.5	38.3	58.2
E	34.0	17.1	35.9	59.2
G	56.4	36.5	60.3	63.7
H	43.4	29.1	37.2	57.8
I	27.7	19.4	20.2	52.7
L	30.3	18.0	27.7	54.6
K	37.5	18.5	36.6	58.8
M	32.8	19.6	29.5	57.0
F	34.3	23.8	26.8	54.1
P	47.8	42.4	25.5	53.1
S	47.3	32.2	36.8	61.1
T	41.8	27.6	27.5	60.0
W	38.2	26.9	29.6	61.2
Y	37.0	25.6	31.6	55.5
V	29.1	19.8	21.9	52.3

The mean absolute error (MAE) of each amino acid for *ψ *angle after two iterations, using six clusters with EM clustering.

The per-residue predictive accuracy of both secondary structure and dihedral clusters based on the SCOP classification of the protein chains is analysed in table 8. Unsurprisingly, residues in all-*α *proteins are predicted particularly well, while the prediction of residues in all-*β *proteins is less accurate. However, the secondary structure prediction of all-*β *proteins is more accurate than the prediction of strand residues shown in table 1. Notably, the predictive accuracy of residues in mixed *α *- *β *proteins is similar to the overall predictive accuracy for secondary structure and dihedral angles, shown in table 1 and table 2, respectively. Residues in *α*/*β *proteins are predicted slightly more accurately than residues in *α *+ *β *proteins.

**Table 8:** Per-residue predictive accuracy based on the SCOP classification of proteins in CB513 dataset.

CB513			
**SCOP class**	**SS pred (%)**	**DihPred3 (%)**	**DihPred7 (%)**

all-*α*	83.6	84.2	67.3
all-*β*	76.4	77.8	48.3
*α*/*β*	81.6	82.2	61.0
*α *+ *β*	79.2	81.4	58.4
Other	75.9	77.8	53.3
All residues	80.0	81.2	58.3

The second column shows the secondary structure predictions while columns three and four show the dihedral prediction using three and seven clusters, respectively.

From table 1, it is clear that the secondary structure prediction improves significantly after the first iteration when the predicted dihedral angles from the initial run (PSSM-only) are used. The subsequent iterations have no impact on the prediction results. Similarly, there is significant improvement in the dihedral prediction after the second iteration when we use the predicted secondary structures from first iteration. Therefore, we use only the iterations that improve the predictions significantly to train DISSPred using PDB-Select25 dataset, i.e. the first iteration for dihedral prediction and the third iteration for both secondary structure and dihedral prediction are omitted, because their results do not improve the predictions of the subsequent iterations. The new design makes the training process faster and, most importantly, it saves time predicting new structures. Table 9 shows the results for secondary structure prediction using PDB-Select25 dataset. The models are trained on one subset and tested using the other. Since no chain in PDB-Select25 has a sequence similarity over 25% with another chain in the dataset, the predictions are independent. The overall accuracy is identical for both subsets. Models trained on subset one predict helical and coil residues slightly better the models trained on subset two, whereas they predict the strand residues slightly worse. Finally, table 10 shows the results for dihedral predictions on PDB-Select25 dataset. The predictive accuracy for small number of clusters is similar to the achieved accuracy using cross validation (table 4), whereas when the number of clusters increases, the accuracy decreases significantly. This suggests that the partition may depend strongly on the dataset used to create the dihedral clusters. Nevertheless, despite limited accuracy, the dihedral prediction can be used to enhance secondary structure prediction (table 9).

**Table 9:** Secondary structure prediction on PDB-Select25 dataset.

PDB-Select25		
**Measure**	**Subset1**	**Subset2**

Q_3 _(%)	79.7	79.7
ErrSig	0.24	0.24
Q_*H *_(%)	82.3	82.6
Q_*E *_(%)	71.9	71.3
Q_*C *_(%)	81.8	82.1
C_*H*_	0.76	0.76
C_*E*_	0.69	0.69
C_*C*_	0.62	0.62
Info	0.43	0.43

The second column shows the predictions for subset1 when SVMs are trained using subset2 and the converse is shown at the third column. Info is a measure of the per-residue information content [4].

**Table 10:** Dihedral prediction on PDB-Select25 dataset.

PDB-Select25				
	**Subset1**		**Subset2**	

**NC**	**EM (%)**	**k-Means (%)**	**EM (%)**	**k-Means (%)**

2	82.9	83.0	82.5	83.1
3	79.0	79.0	78.9	79.1
4	74.6	71.5	74.2	72.1
5	59.8	57.3	59.5	57.5
6	58.9	53.4	58.4	53.5
7	48.6	48.0	48.5	47.8

Columns two and three show the predictions for subset1 when SVMs are trained using subset2 and the converse is shown in columns four and five.

### DISSPred server

Our method is available online at http://comp.chem.nottingham.ac.uk/disspred/. DISSPred is written in Perl using a CGI interface. Only FASTA files are accepted as input or compressed archives, containing FASTA files. The user can choose the preferred clustering algorithm and the number of clusters. For each input file, one output file is created that contains the amino acid type, the amino acid number in the sequence, the predicted secondary structure, the predicted dihedral cluster and the *ϕ *and *ψ *values of the predicted cluster centre. The output files, together with the log files, are sent to the user by e-mail after the calculations are completed. Table 11 shows DISSPred's prediction accuracy, for different subsets of the dataset provided by the EVA secondary structure prediction server [54], compared with other secondary structure prediction servers: PSIPRED [6], PHDpsi [65], PROFsec [54], SAM-T99 sec [66], PROFking [9] and Prospect [67]. Note that the results are not independent predictions, since some of the sequences in EVA dataset are homologous with some sequences in PDB-Select25 dataset, which was used to train DISSPred.

**Table 11:** Performance of DISSPred and other secondary structure predictors on EVA dataset.

EVA subsets - Q_3_(%)				
**Method**	**EVA1**	**EVA2**	**EVA4**	**EVA6**

DISSPred	81.7	81.9	81.9	82.0
PSIPRED	76.8	77.4	77.3	77.8
PHDpsi	73.4	74.3	74.3	75.0
PROFsec	75.5	76.2	76.4	76.7
SAM-T99 sec	77.2	77.2	77.1	N/A
PROFking	71.6	71.7	N/A	N/A
Prospect	71.1	N/A	N/A	N/A

The results for the other methods were obtained from EVA secondary structure prediction server.

## Conclusions

Using predicted secondary structure and dihedral angles, our method improves the predictive accuracy of both secondary structure and dihedral angle prediction in an iterative process using SVMs. The achieved secondary structure Q_3 _accuracy of 80% on a set of 513 non-redundant proteins shows that our method is more accurate than other secondary structure prediction methods. The dihedrally-enhanced secondary structure prediction method significantly improved the predictive accuracy of helical and extended residues. Moreover, the prediction of dihedral angles is more accurate than other multi-state dihedral prediction methods and achieves a MAE comparable to the reported MAE of Real-SPINE 2.0 and 3.0 [41,42], a real-value dihedral predictor. The online version of DISSPred was trained using the larger PDB-Select25 dataset. We are currently investigating the use of predicted dihedral angles as constraints for molecular dynamics simulations and together with the secondary structure predictions to facilitate predictions of protein tertiary structure. Finally, we are working on enhancing the prediction of tight turns in proteins using predicted dihedral angles.

## Authors' contributions

PK carried out the experiments and wrote the manuscript. JDH conceived the study and assisted in writing the manuscript. Both authors read and approved the final manuscript for publication.

## Supplementary Material

Additional file 1 **Cluster centroids and standard deviation for each cluster**. The cluster centres with the standard deviation of each cluster are shown for all the different partitions of the *ϕ *- *ψ *space are shown using EM and k-Means clustering. Click here for file

Additional file 2 **Clustering and secondary structure distribution in every cluster**. All the different partitions of the *ϕ *- *ψ *space are shown using EM and k-Means clustering as well as the distribution of secondary structure element in each cluster. Click here for file

Additional file 3 **Secondary structure prediction in every cluster before and after using additional dihedral information**. The impact of additional dihedral information on the secondary structure prediction in every cluster is presented. Click here for file

Additional file 4 **The MAE and Q_30 _after the second iteration of DISSPred using EM clustering**. The mean absolute errors (MAEs) and the percentage of predicted dihedral angles within 30° of the real value (Q_30_) for both backbone dihedral angles *ϕ *and *ψ *after two iterations of our method using EM clustering. In bold are the best results in every case. Click here for file
