# Supplementary material for: Prediction of backbone dihedral angles and protein secondary structure using support vector machines
Source: BMC Bioinformatics. 2009 Dec 22;10:437. doi: 10.1186/1471-2105-10-437 (PMC2811710; doi:10.1186/1471-2105-10-437)
Supplement: Additional file 1 — Cluster centroids and standard deviation for each cluster. The cluster centres with the standard deviation of each cluster are shown for all the different partitions of the ϕ - ψ space are shown using EM and k-Means clustering. [file 1471-2105-10-437-S1.PDF]

Additional file 1

Clustering Algorithm: EM

Below are the centoids and standard deviation of every cluster using Expectation Maximisation and number of clusters from 2 to twelve.

Clusters: 2				
Cluster	ϕ	ψ	SD_ϕ	SD_ψ
0	-92.5	137.9	58.4	32.6
1	-60.5	-29.2	43.4	22.7

Clusters: 3				
Cluster	ϕ	ψ	SD_ϕ	SD_ψ
0	-70.5	-32.8	17.7	18.6
1	83.2	66.1	34.2	84.3
2	-106.1	135.8	30.7	31.2

Clusters: 4				
Cluster	ϕ	ψ	SD_ϕ	SD_ψ
0	-106.1	135.8	30.8	31.1
1	-70.5	-32.8	17.8	18.6
2	103.9	187.8	43.5	36.4
3	75.3	17.1	24.6	32.1

Clusters: 5				
Cluster	ϕ	ψ	SD_ϕ	SD_ψ
0	-62.6	-40.2	7.4	9.3
1	-71.5	140.1	13.8	16.1
2	-119.3	138.6	24.5	28.7
3	81.9	66.0	35.9	84.7
4	-89.6	-12.4	22.3	26.9

Clusters: 6				
Cluster	ϕ	ψ	SD_ϕ	SD_ψ
0	-120.7	138.8	23.8	28.7
1	-62.6	-40.2	7.4	9.3
2	-89.1	-12.6	22.7	27.4
3	75.1	17.0	24.8	32.2
4	100.4	186.9	47.8	37.6
5	-72.8	139.6	14.6	17.1

Clusters: 7				
Cluster	ϕ	ψ	SD_ϕ	SD_ψ
0	-135.0	148.8	18.7	17.6
1	-62.5	-40.4	7.3	9.1
2	-88.2	-14.7	22.6	25.4
3	75.1	17.0	24.8	32.2
4	101.7	187.0	46.0	37.2
5	-107.3	131.1	23.9	33.1
6	-70.2	141.2	12.7	14.4

Clusters: 8				
Cluster	ϕ	ψ	SD_ϕ	SD_ψ
0	-80.6	-16.2	12.9	13.5
1	97.8	187.3	51.6	37.9
2	-72.1	142.0	13.5	18.4
3	-62.2	-41.0	7.5	8.7
4	-100.2	7.6	27.1	47.0
5	75.2	16.9	24.7	32.1
6	-134.0	153.2	20.8	24.8
7	-111.7	128.8	16.6	17.7

Clusters: 9				
Cluster	ϕ	ψ	SD_ϕ	SD_ψ
0	-144.7	157.8	15.1	14.2
1	-78.3	-17.2	11.4	12.2
2	-95.8	-10.7	25.6	32.9
3	-62.2	-41.1	7.3	8.5
4	102.8	187.2	44.7	36.8
5	-113.5	132.3	17.7	16.8
6	-107.2	130.3	28.2	45.1
7	75.2	17.0	24.7	32.2
8	-70.1	142.0	12.1	15.7

Clusters: 10				
Cluster	ϕ	ψ	SD_ϕ	SD_ψ
0	-143.4	157.2	15.6	14.2
1	-71.7	-23.1	8.6	10.3
2	-108.2	90.1	28.1	27.2
3	-61.7	-42.1	7.1	7.7
4	102.7	187.2	44.8	36.8
5	-114.1	131.5	16.8	15.8
6	-107.8	151.0	28.3	38.4
7	75.1	17.0	24.8	32.2
8	-71.1	141.8	12.8	16.0
9	-92.9	-12.6	23.7	29.0

Clusters: 11				
Cluster	ϕ	ψ	SD_ϕ	SD_ψ
0	-71.1	-50.7	29.8	16.4
1	-97.8	-1.6	14.7	15.7
2	75.5	17.4	24.4	31.6
3	102.9	187.0	44.6	37.0
4	-114.1	131.5	16.9	16.0
5	-104.2	142.6	28.5	40.4
6	-70.8	-25.2	9.1	11.1
7	-70.8	141.7	12.5	15.9
8	-112.1	75.7	26.3	58.2
9	-143.8	157.4	15.5	14.4
10	-61.7	-42.3	6.4	7.1

Clusters: 12				
Cluster	ϕ	ψ	SD_ϕ	SD_ψ
0	-152.7	160.0	11.9	17.2
1	-96.2	141.0	20.7	36.9
2	-71.1	-50.7	29.9	16.5
3	-61.7	-42.3	6.4	7.1
4	-126.7	140.9	12.4	17.1
5	75.5	17.4	24.4	31.5
6	-115.2	81.1	27.2	59.2
7	101.6	186.5	46.0	37.5
8	-104.6	124.9	12.4	12.6
9	-97.7	-1.6	14.8	15.9
10	-70.5	141.6	12.8	15.1
11	-70.7	-25.4	9.1	11.1

Clustering Algorithm: KMeans

Below are the centoids and standard deviation of every cluster using k-Means clustering algorithm and number of clusters from 2 to twelve.

Clusters: 2				
Cluster	ϕ	ψ	SD_ϕ	SD_ψ
0	-58.2	-27.0	46.6	25.9
1	-96.4	140.8	53.6	28.8

Clusters: 3				
Cluster	ϕ	ψ	SD_ϕ	SD_ψ
0	84.3	67.2	33.5	82.6
1	-105.8	138.7	31.0	26.6
2	-71.3	-31.4	19.6	20.9

Clusters: 4				
Cluster	ϕ	ψ	SD_ϕ	SD_ψ
0	-64.4	-39.3	12.7	12.1
1	-105.6	140.3	31.1	24.4
2	-100.7	6.1	19.7	24.5
3	84.3	67.5	33.6	82.4

Clusters: 5				
Cluster	ϕ	ψ	SD_ϕ	SD_ψ
0	-64.3	-39.3	12.2	12.0
1	-74.9	139.9	23.9	26.7
2	-100.4	4.5	19.3	23.5
3	86.0	58.2	33.4	75.2
4	-129.9	141.5	17.5	25.4

Clusters: 6				
Cluster	ϕ	ψ	SD_ϕ	SD_ψ
0	-63.9	-39.6	11.6	11.7
1	-108.5	115.5	18.1	18.9
2	-99.1	-0.0	19.0	20.2
3	86.3	55.1	33.6	72.8
4	-136.1	156.2	17.5	19.2
5	-67.2	149.7	27.9	23.7

Clusters: 7				
Cluster	ϕ	ψ	SD_ϕ	SD_ψ
0	-61.9	-42.2	10.5	9.7
1	-108.5	118.4	17.5	17.0
2	-109.7	20.0	19.0	20.7
3	86.4	55.1	33.6	72.7
4	-136.8	157.1	17.4	19.2
5	-66.7	149.9	28.0	23.9
6	-83.4	-18.4	16.1	13.5

Clusters: 8				
Cluster	ϕ	ψ	SD_ϕ	SD_ψ
0	-62.0	-42.3	9.6	9.6
1	-111.3	117.0	17.1	17.1
2	-109.1	18.6	18.8	20.3
3	75.8	17.7	24.7	31.4
4	-73.3	145.3	14.8	18.6
5	104.6	188.8	42.3	35.0
6	-82.9	-18.8	15.9	13.3
7	-137.0	157.6	17.4	19.1

Clusters: 9				
Cluster	ϕ	ψ	SD_ϕ	SD_ψ
0	-108.2	8.4	17.4	18.4
1	-120.3	131.8	14.1	12.1
2	-100.0	97.1	20.3	17.9
3	75.8	17.7	24.7	31.4
4	-73.1	145.7	14.6	18.0
5	104.6	188.8	42.3	35.0
6	-77.7	-21.3	13.5	12.1
7	-141.3	164.5	17.5	18.8
8	-61.7	-43.3	9.8	9.1

Clusters: 10				
Cluster	ϕ	ψ	SD_ϕ	SD_ψ
0	-73.4	-18.7	9.7	9.8
1	-120.7	132.2	14.0	12.2
2	-106.1	-39.2	16.7	18.1
3	75.8	17.7	24.7	31.4
4	-73.1	145.9	14.6	17.9
5	104.6	188.8	42.3	35.0
6	-99.9	98.7	19.9	17.8
7	-141.4	164.7	17.5	18.7
8	-61.3	-43.2	8.7	8.2
9	-106.8	12.4	16.8	15.6

Clusters: 11				
Cluster	ϕ	ψ	SD_ϕ	SD_ψ
0	-72.9	-19.5	9.5	9.7
1	-69.2	135.6	13.4	12.7
2	-105.9	-39.8	16.8	18.0
3	75.8	17.7	24.7	31.3
4	-117.2	129.7	13.6	11.3
5	104.6	188.8	42.2	34.9
6	-105.3	88.9	22.6	16.8
7	-144.5	159.4	14.2	16.4
8	-61.2	-43.4	8.7	8.2
9	-105.8	10.6	16.4	14.5
10	-89.8	169.6	15.2	18.9

Clusters: 12				
Cluster	ϕ	ψ	SD_ϕ	SD_ψ
0	-84.7	-6.4	10.6	10.0
1	-69.3	136.3	13.5	12.4
2	-107.3	-41.2	17.0	18.1
3	75.8	17.7	24.7	31.3
4	-118.4	130.4	13.6	11.4
5	104.6	188.8	42.2	34.9
6	-67.2	-31.0	7.5	7.4
7	-145.1	160.0	14.0	16.3
8	-59.3	-46.7	8.7	7.3
9	-114.1	17.5	16.1	16.0
10	-90.4	170.0	15.3	19.0
11	-102.8	92.9	20.9	16.6
