# Supplementary material for: Prediction of backbone dihedral angles and protein secondary structure using support vector machines
Source: BMC Bioinformatics. 2009 Dec 22;10:437. doi: 10.1186/1471-2105-10-437 (PMC2811710; doi:10.1186/1471-2105-10-437)
Supplement: Additional file 2 — Clustering and secondary structure distribution in every cluster. All the different partitions of the ϕ - ψ space are shown using EM and k-Means clustering as well as the distribution of secondary structure element in each cluster. [file 1471-2105-10-437-S2.PDF]

Additional file 2

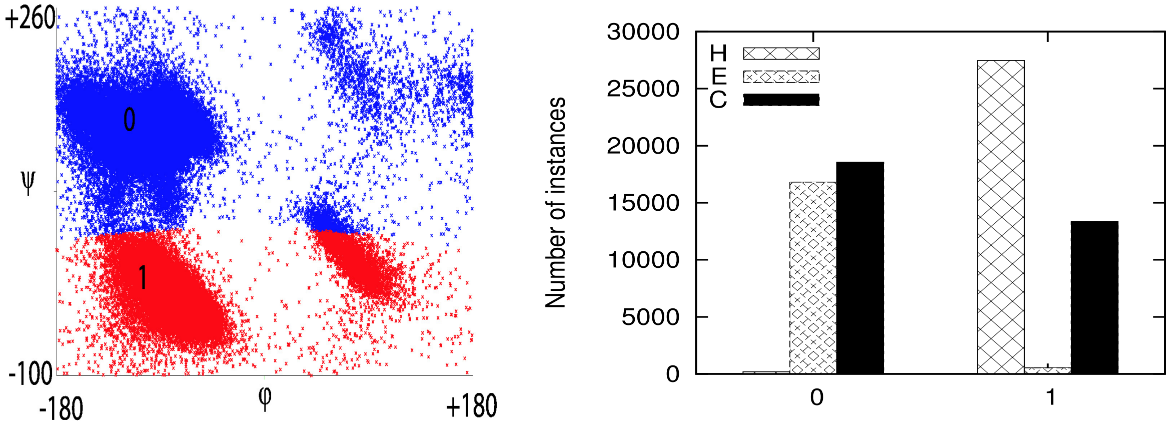

Figure 1: EM - clusters: 2

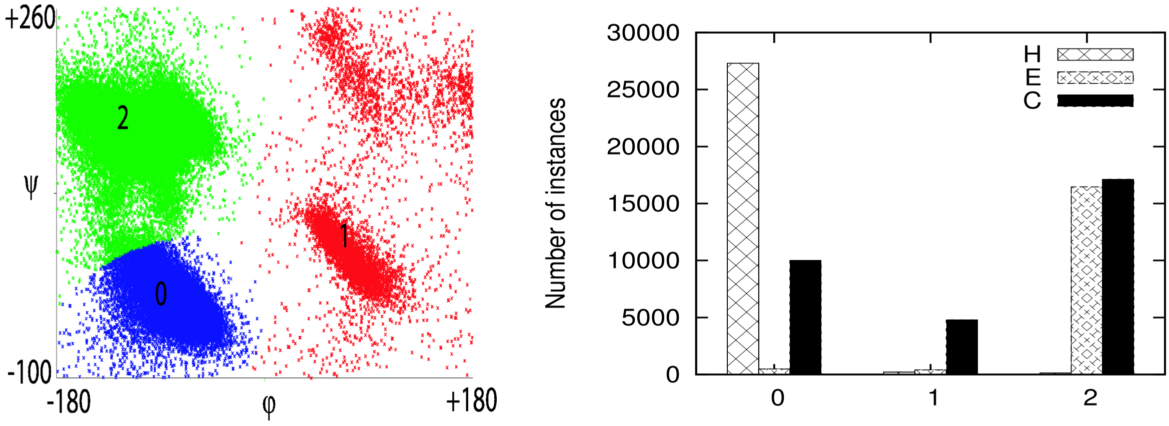

Figure 2: EM - clusters: 3

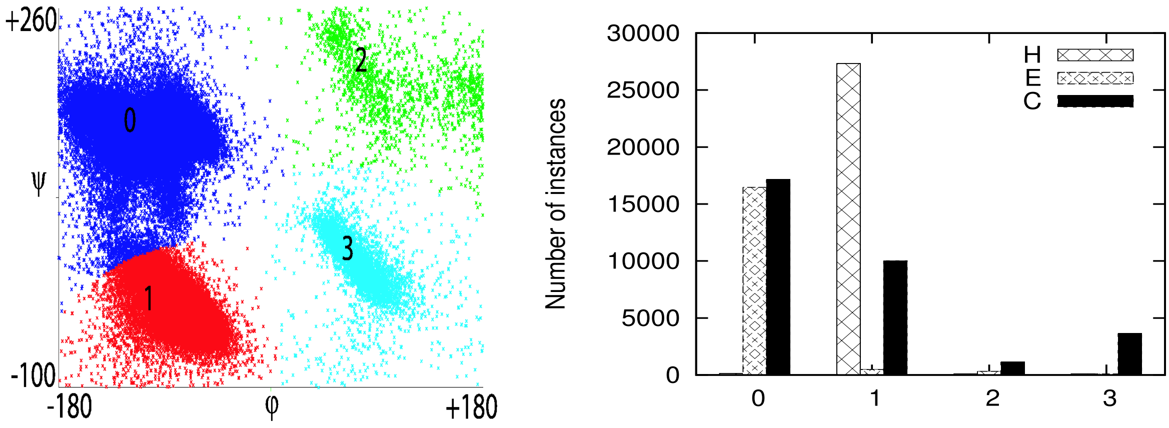

Figure 3: EM - clusters: 4

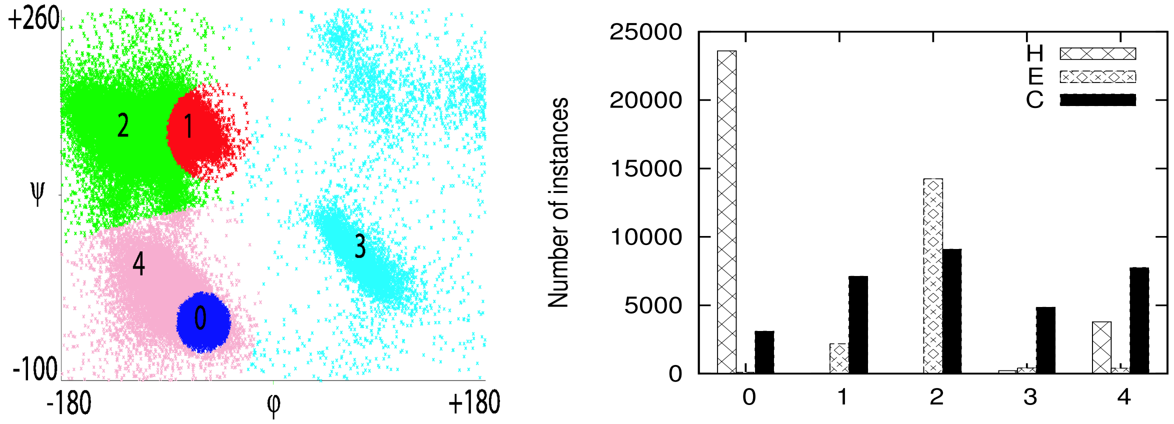

Figure 4: EM - clusters: 5

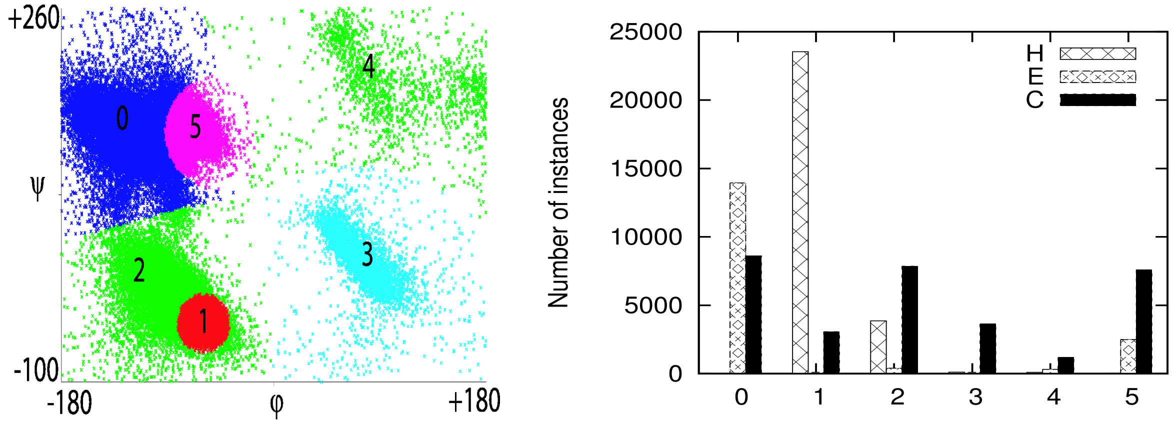

Figure 5: EM - clusters: 6

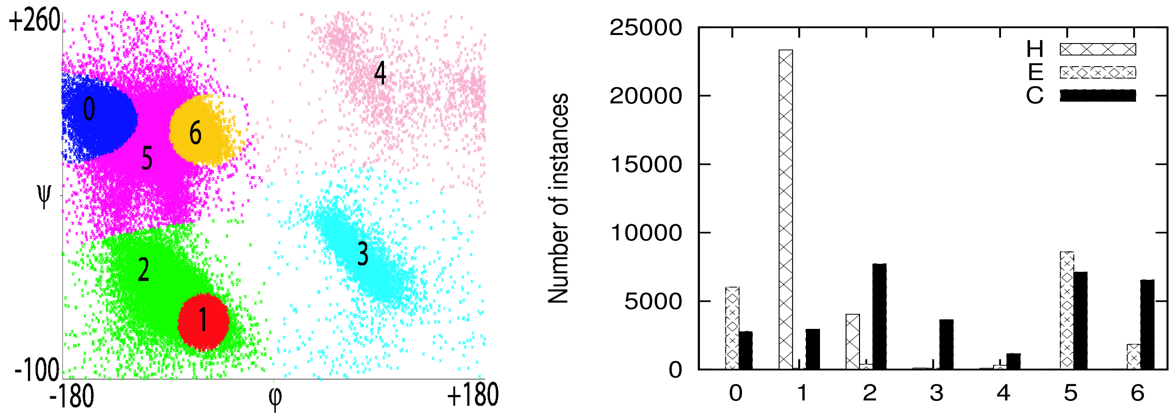

Figure 6: EM - clusters: 7

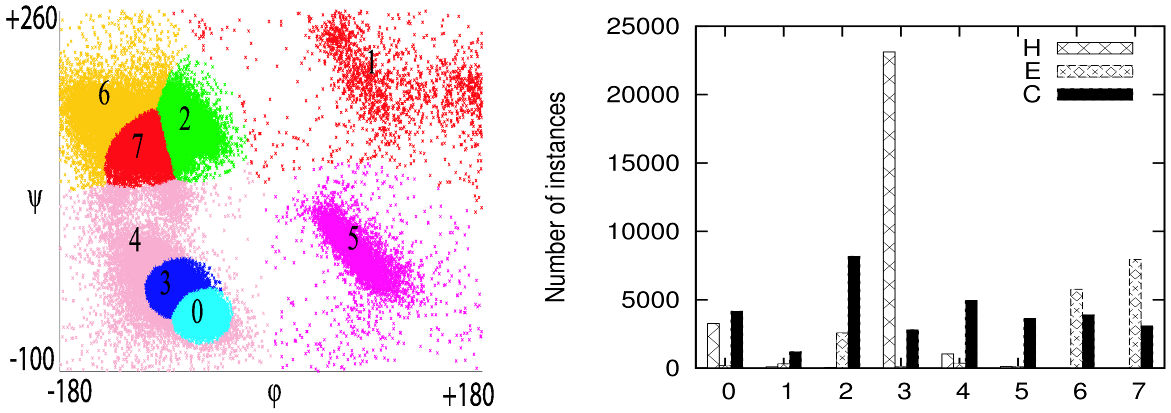

Figure 7: EM - clusters: 8

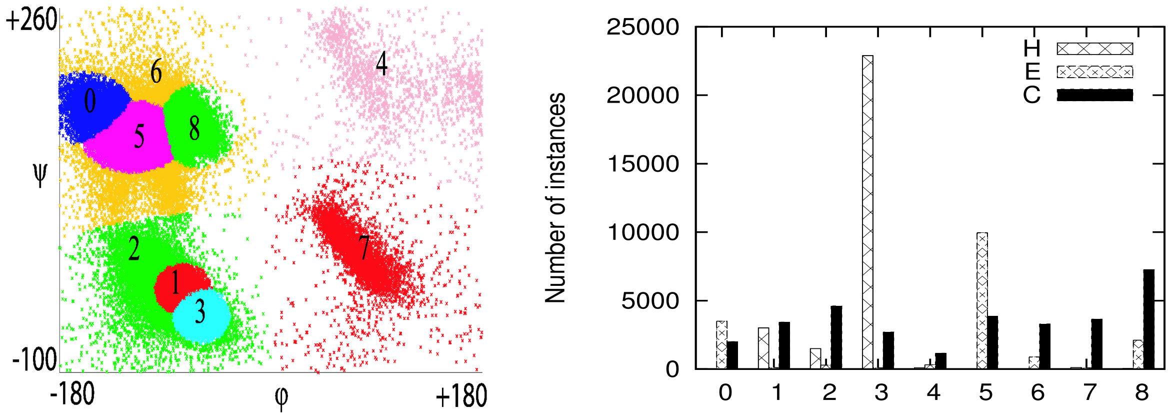

Figure 8: EM - clusters: 9

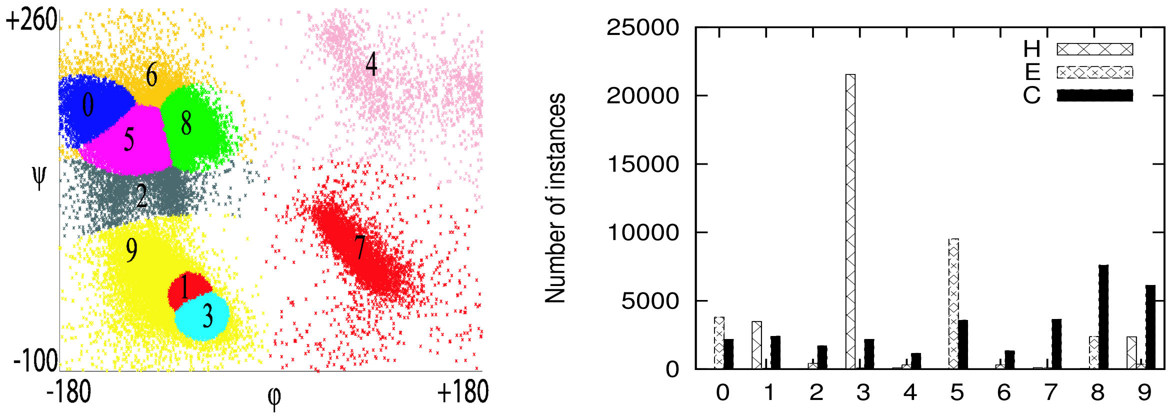

Figure 9: EM - clusters: 10

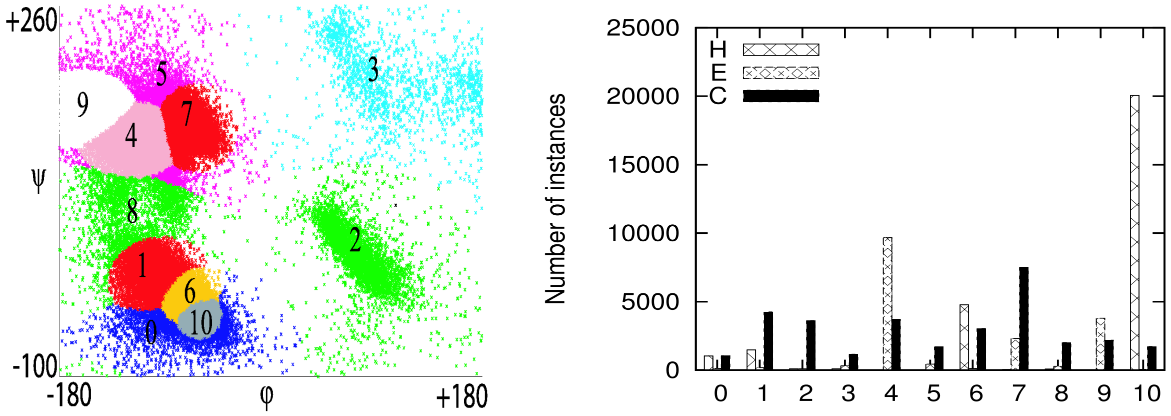

Figure 10: EM - clusters: 11

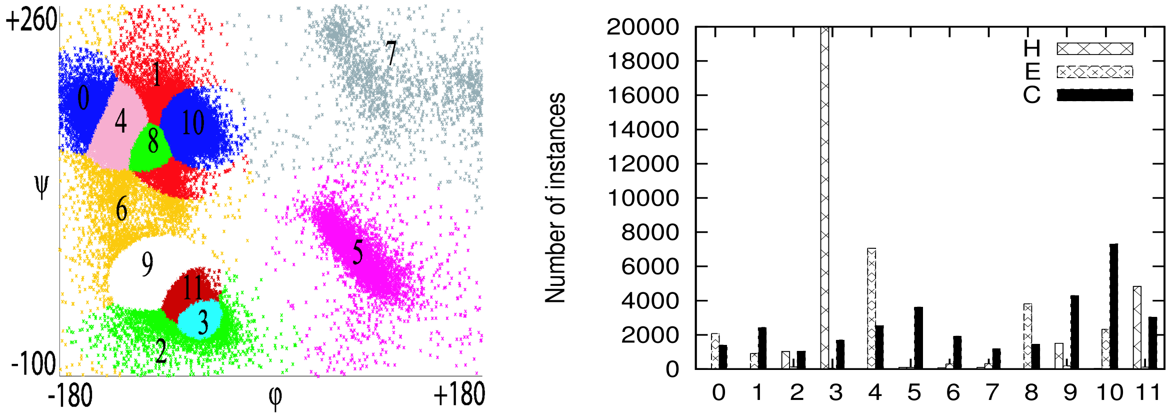

Figure 11: EM - clusters: 12

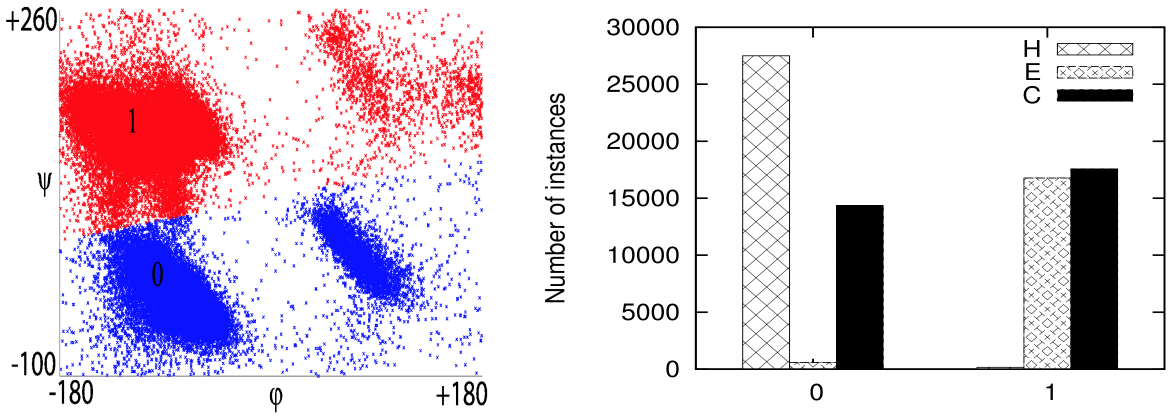

Figure 12: KMeans - clusters: 2

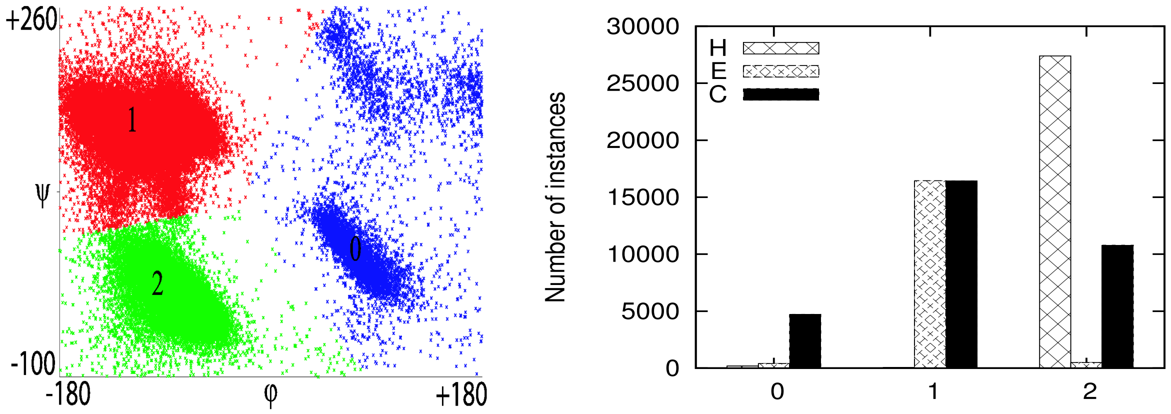

Figure 13: KMeans - clusters: 3

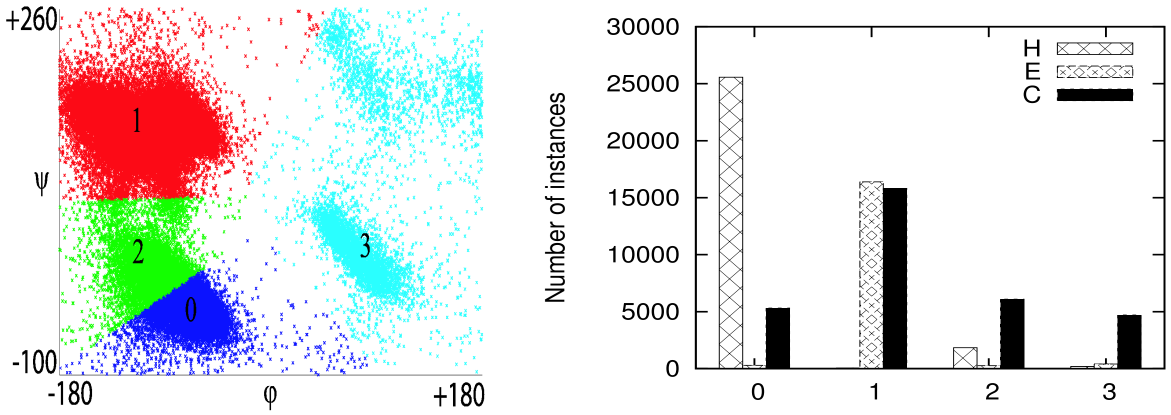

Figure 14: KMeans - clusters: 4

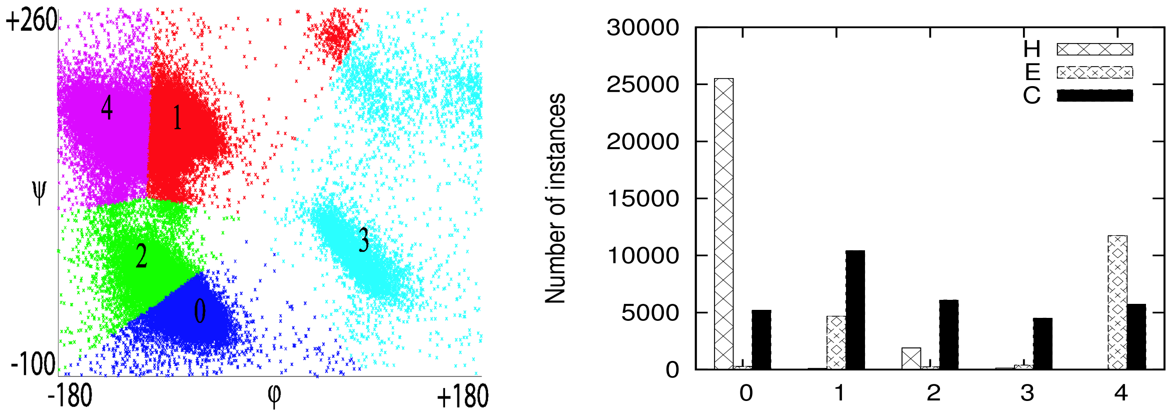

Figure 15: KMeans - clusters: 5

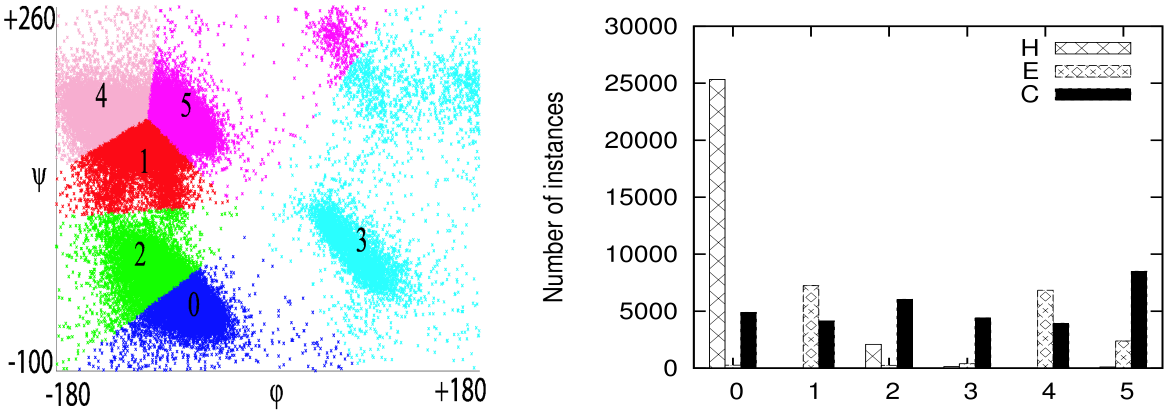

Figure 16: KMeans - clusters: 6

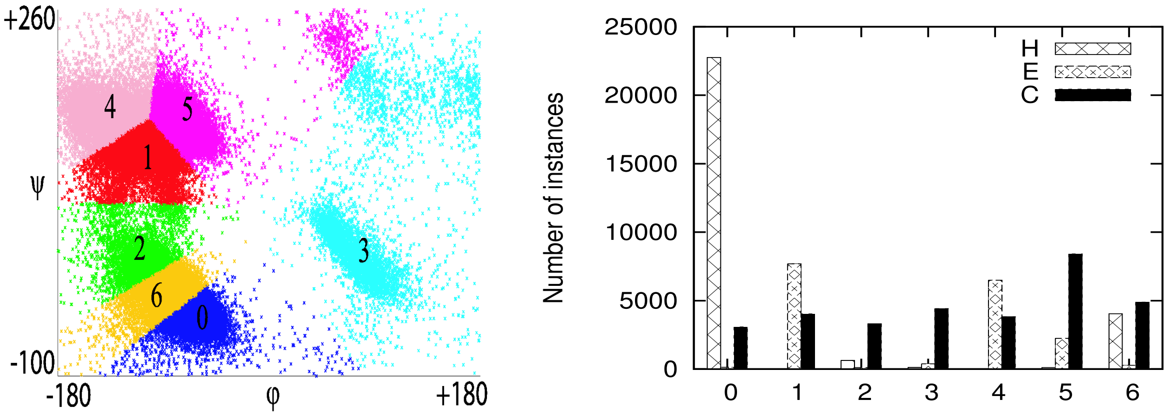

Figure 17: KMeans - clusters: 7

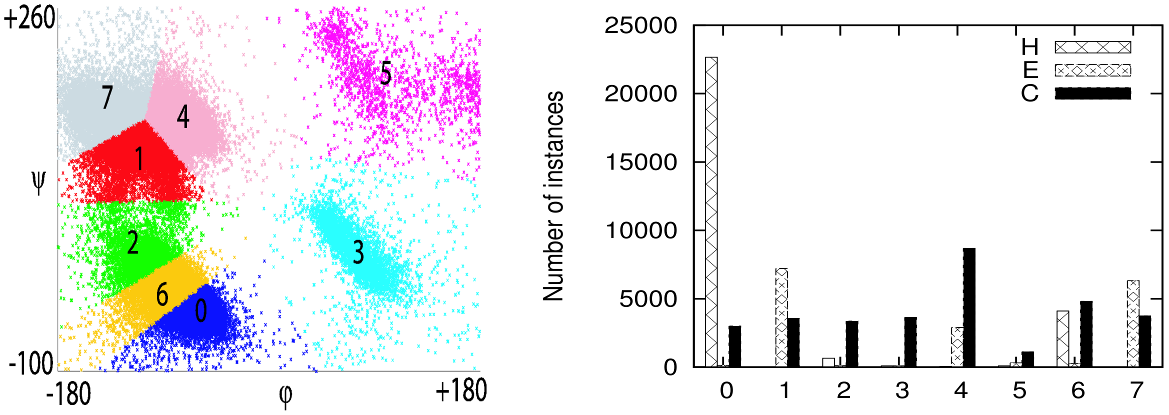

Figure 18: KMeans - clusters: 8

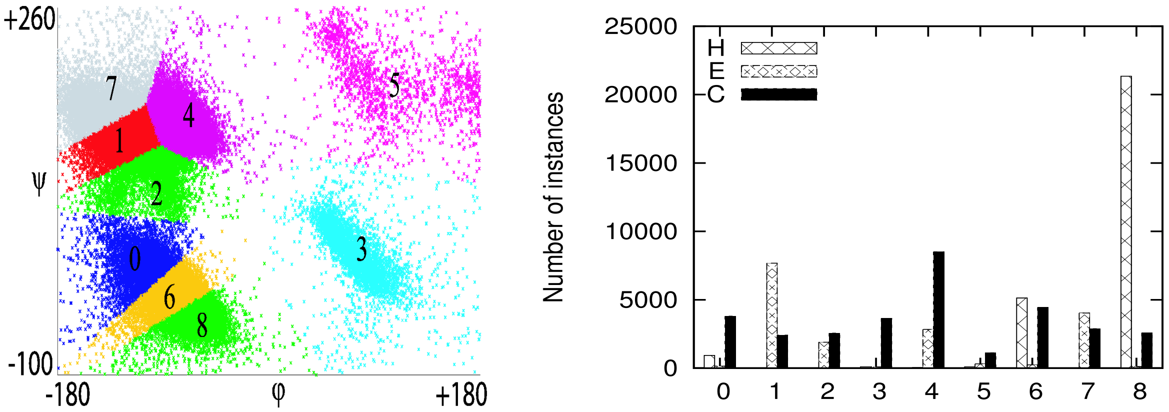

Figure 19: KMeans - clusters: 9

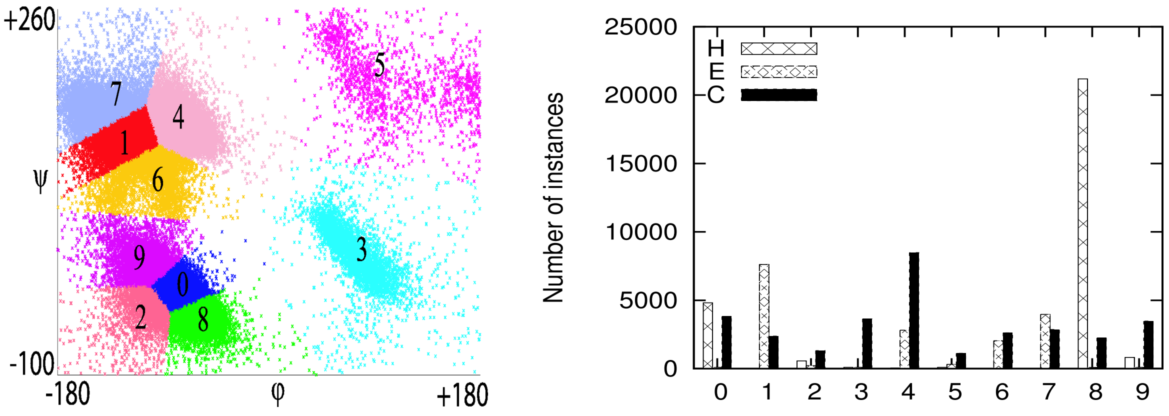

Figure 20: KMeans - clusters: 10

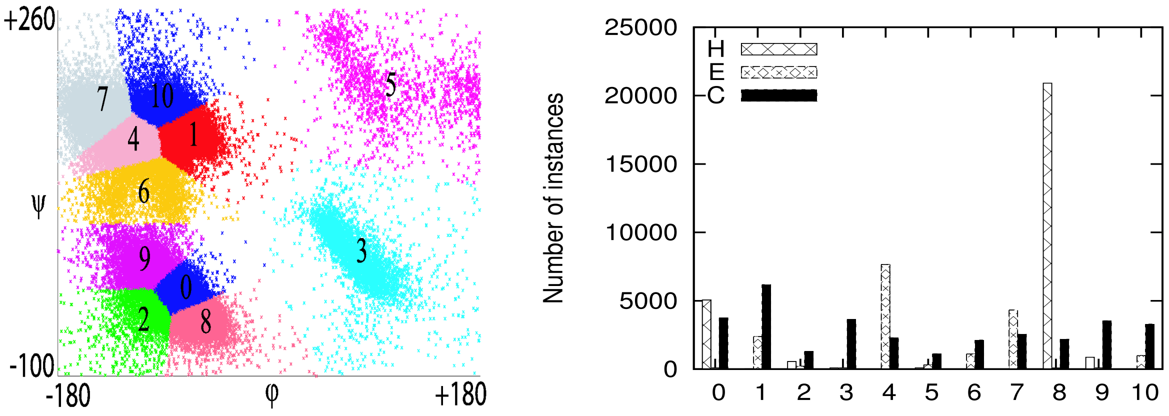

Figure 21: KMeans - clusters: 11

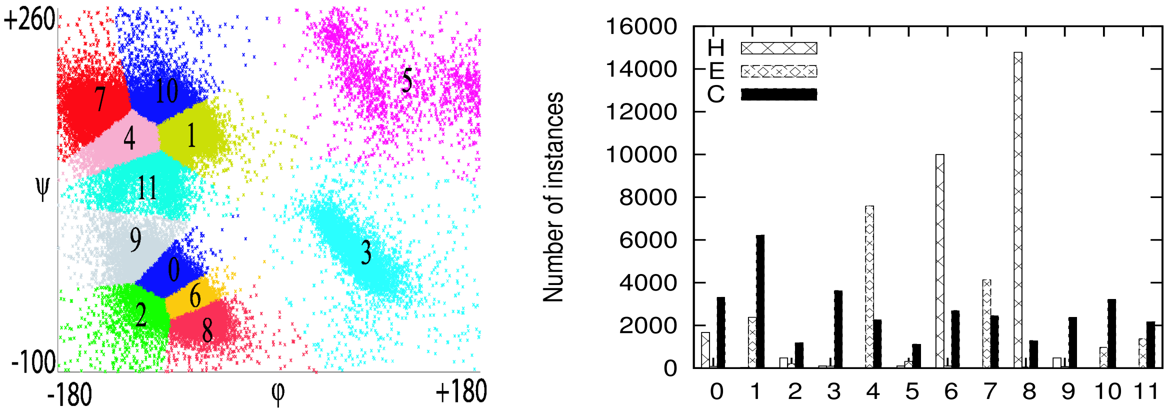

Figure 22: KMeans - clusters: 12
