# Supplementary material for: Prediction of backbone dihedral angles and protein secondary structure using support vector machines
Source: BMC Bioinformatics. 2009 Dec 22;10:437. doi: 10.1186/1471-2105-10-437 (PMC2811710; doi:10.1186/1471-2105-10-437)
Supplement: Additional file 3 — Secondary structure prediction in every cluster before and after using additional dihedral information. The impact of additional dihedral information on the secondary structure prediction in every cluster is presented. [file 1471-2105-10-437-S3.PDF]

Additional file 3

Expectation Maximisation

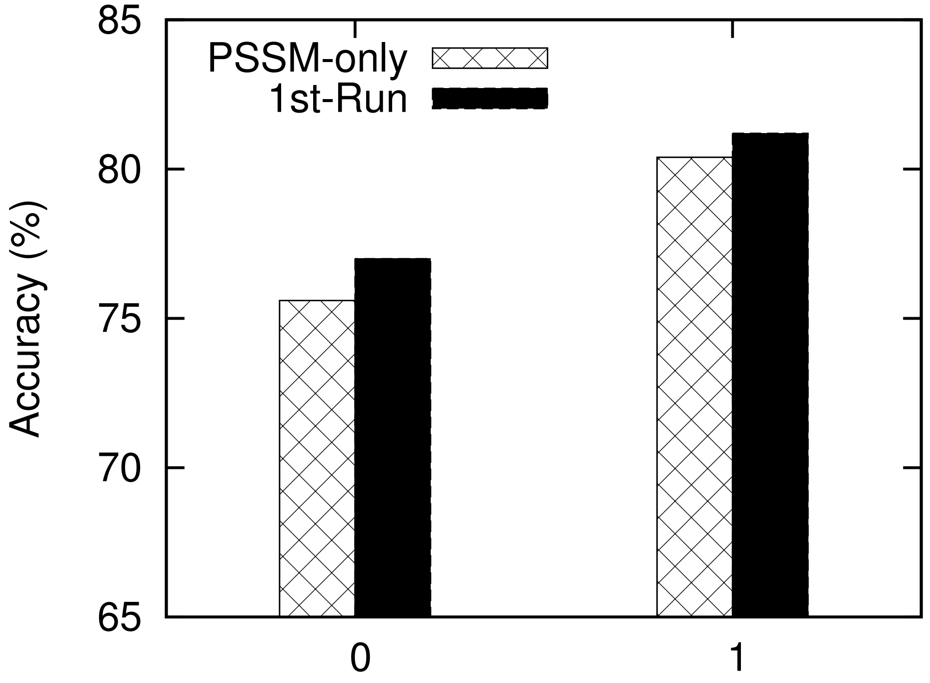

Figure 1: EM - Clusters: 2

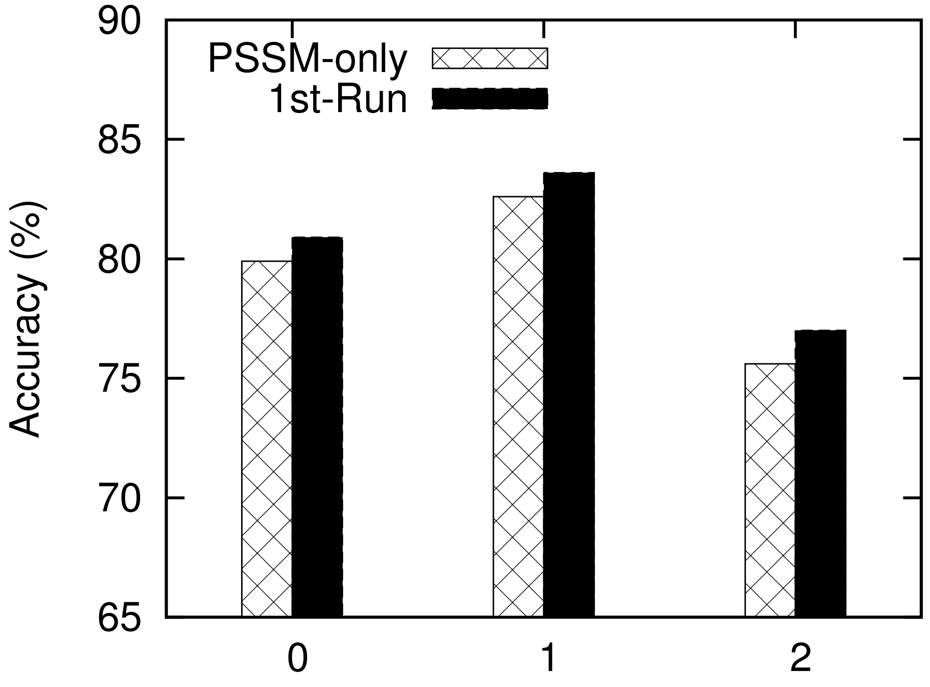

Figure 2: EM - Clusters: 3

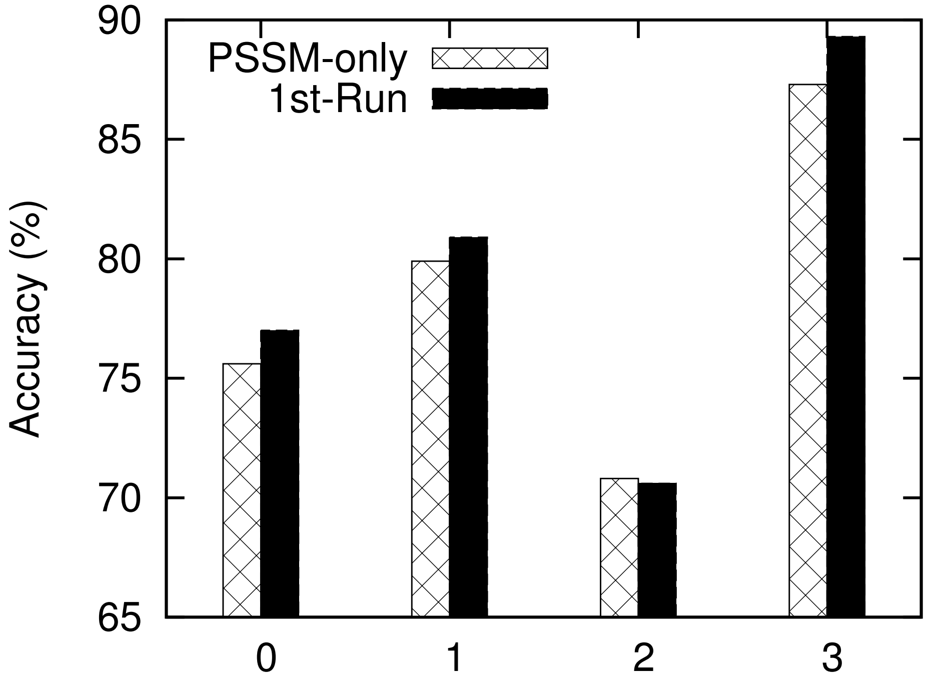

Figure 3: EM - Clusters: 4

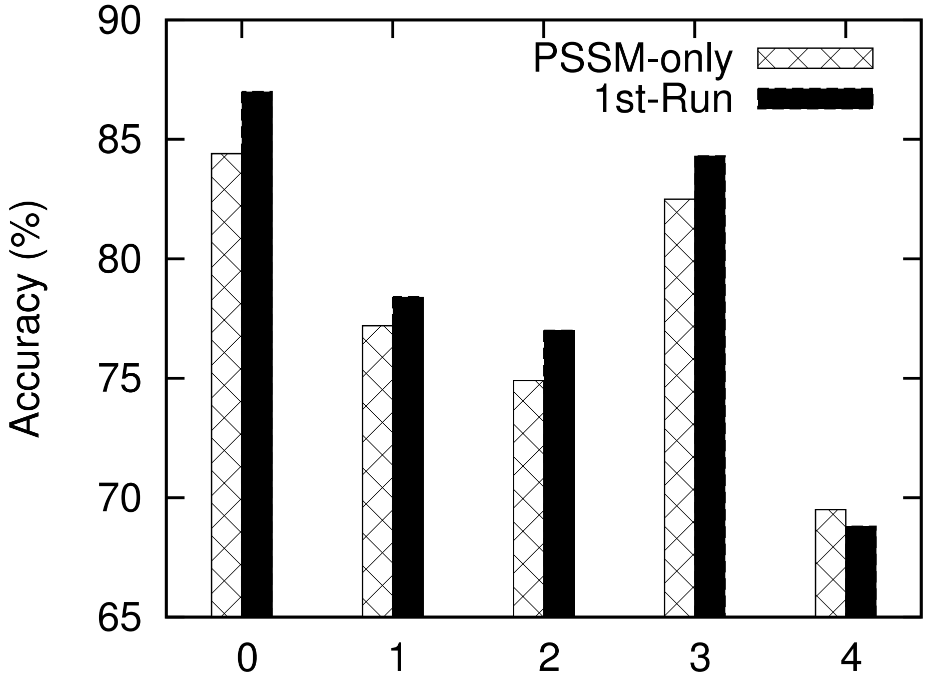

Figure 4: EM - Clusters: 5

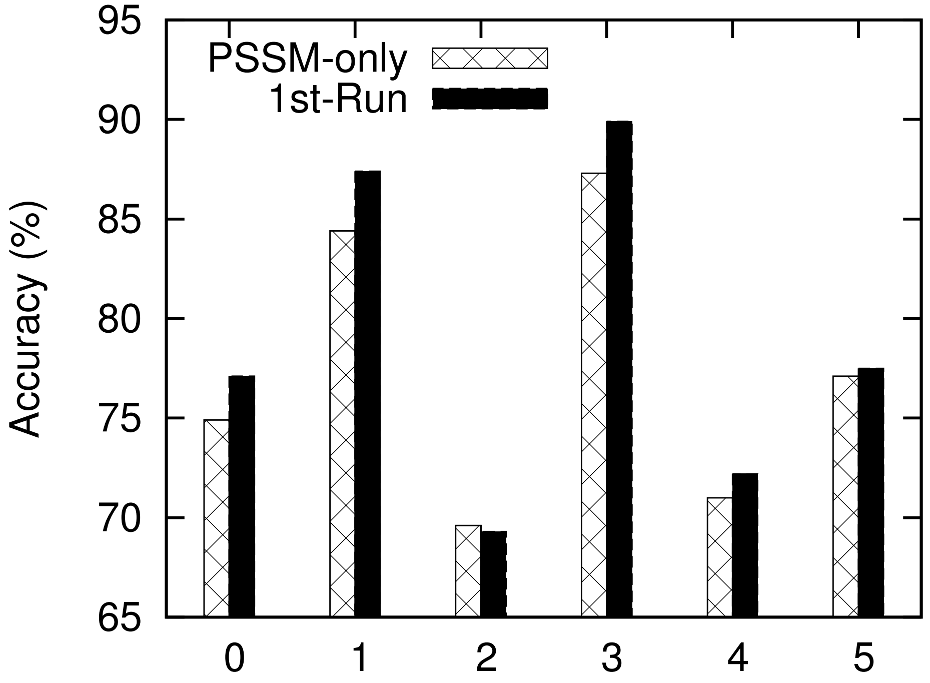

Figure 5: EM - Clusters: 6

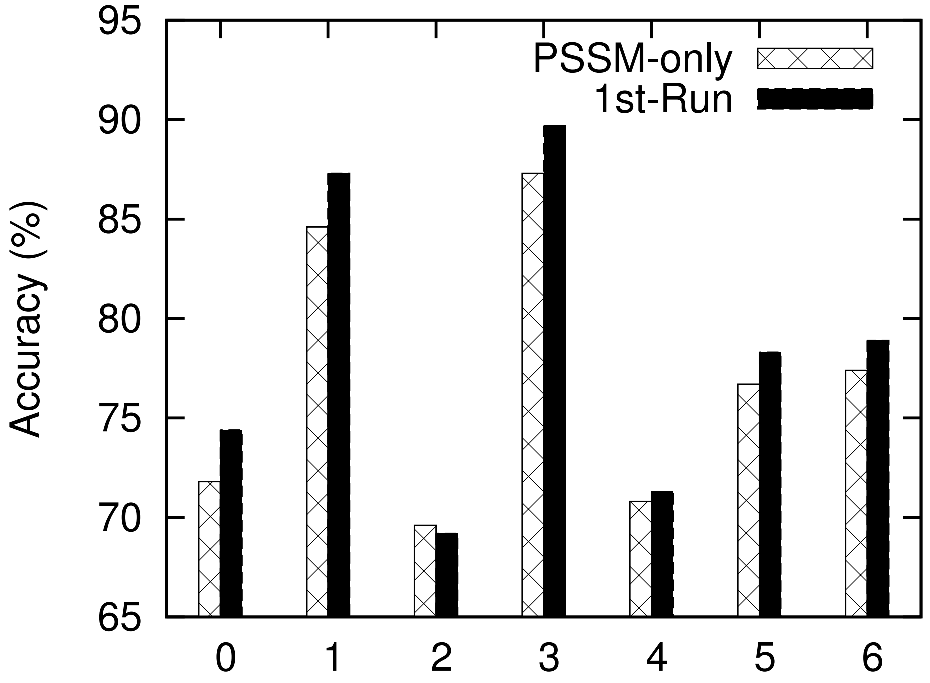

Figure 6: EM - Clusters: 7

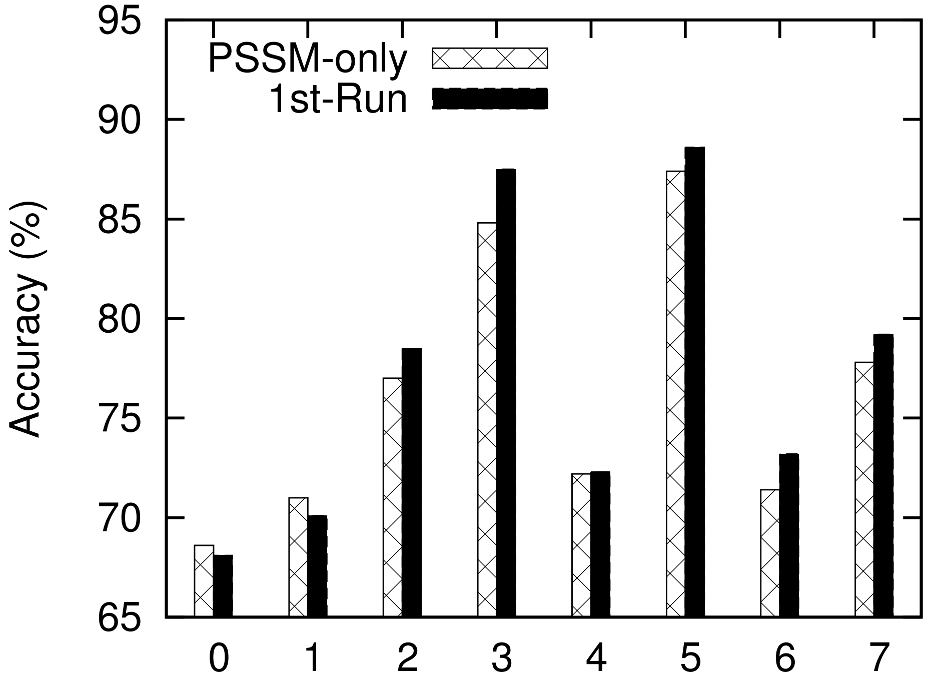

Figure 7: EM - Clusters: 8

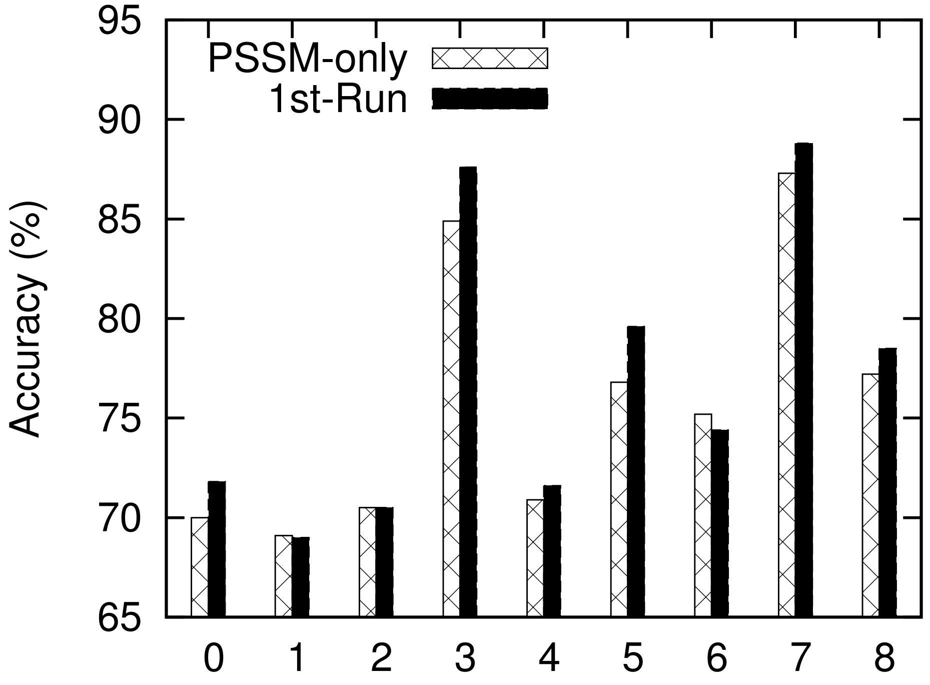

Figure 8: EM - Clusters: 9

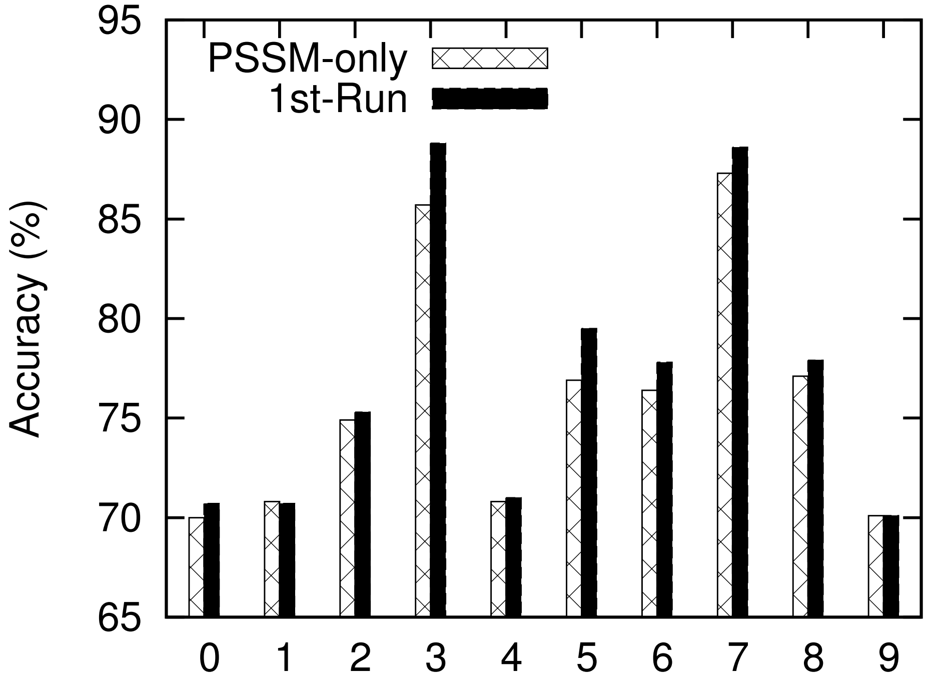

Figure 9: EM - Clusters: 10

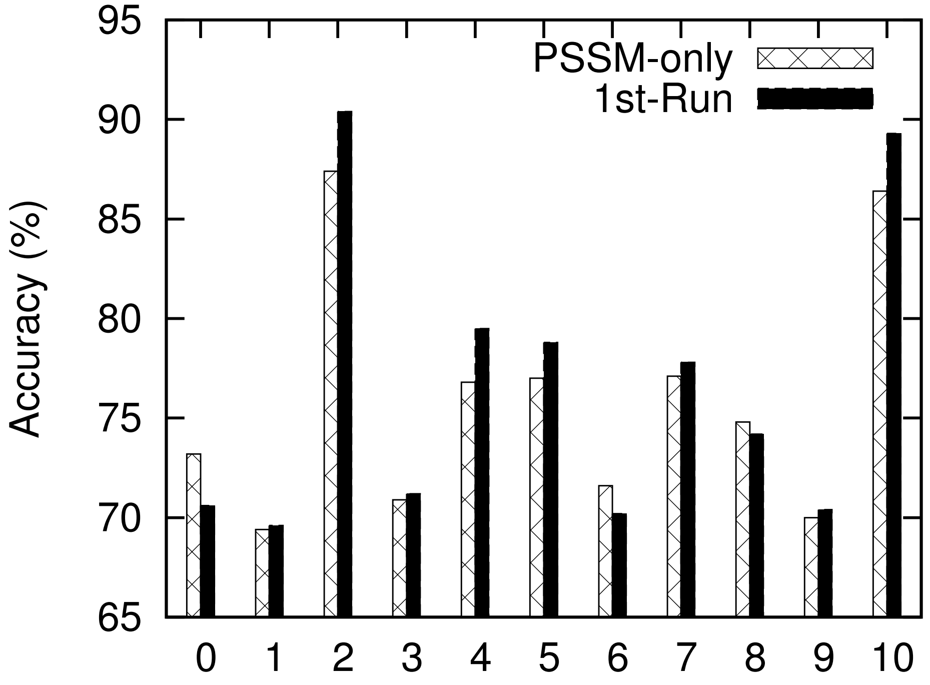

Figure 10: EM - Clusters: 11

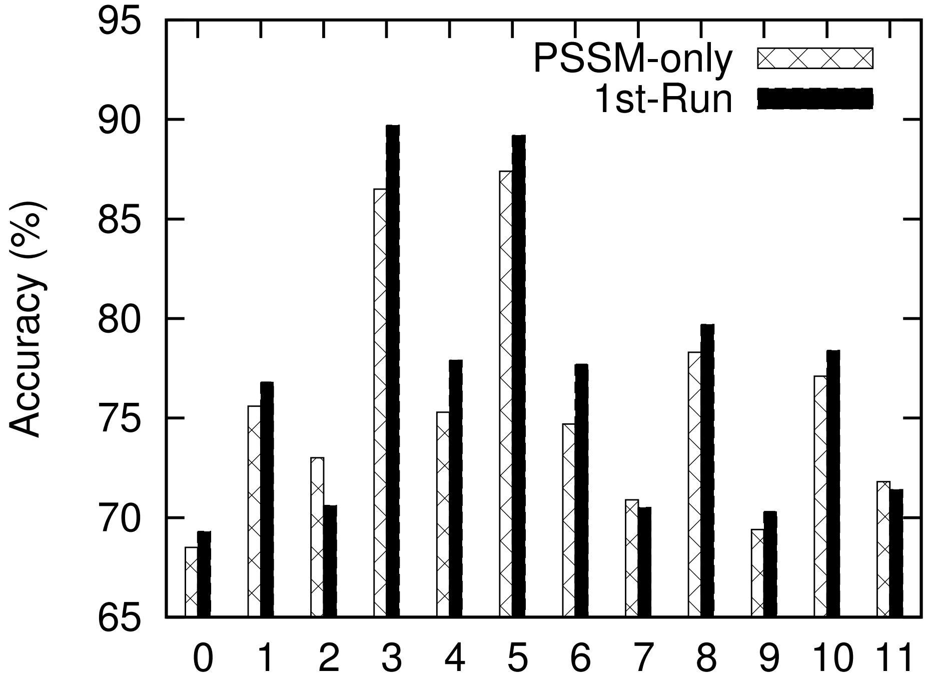

Figure 11: EM - Clusters: 12

k-Means clustering

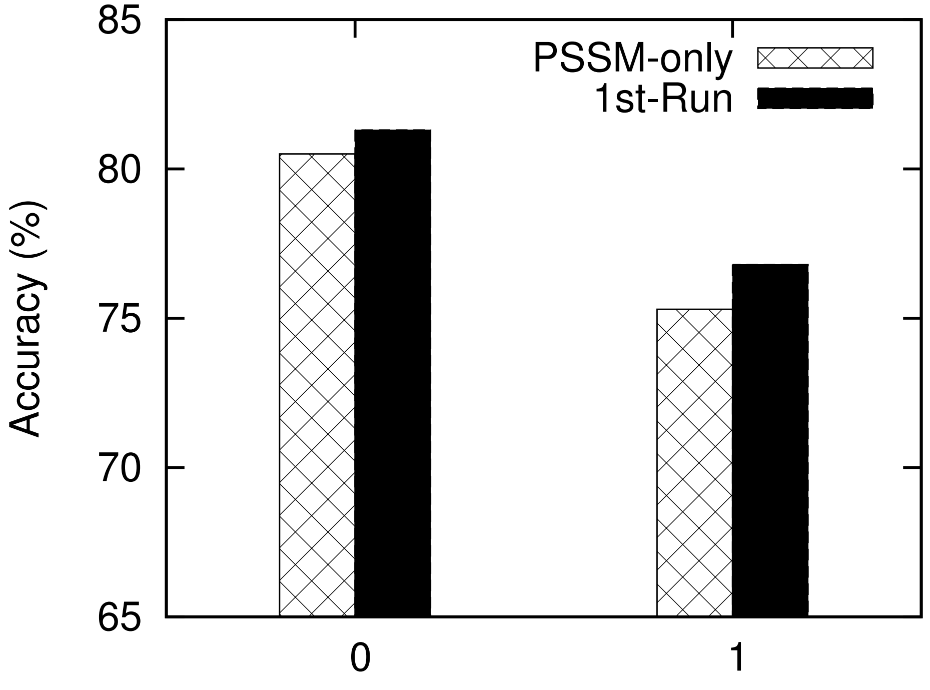

Figure 12: k-Means - Clusters: 2

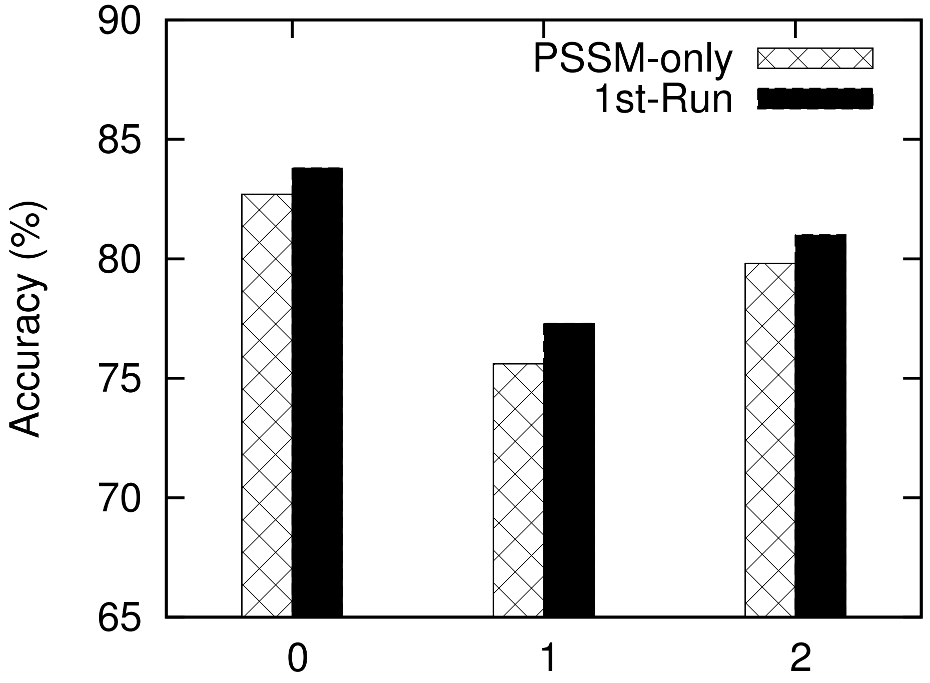

Figure 13: k-Means - Clusters: 3

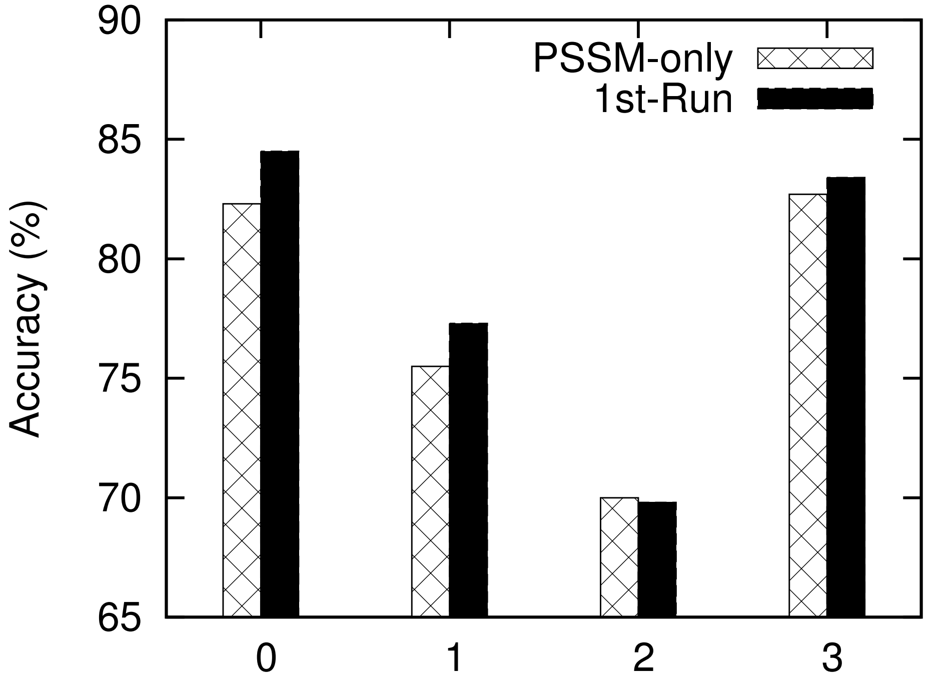

Figure 14: k-Means - Clusters: 4

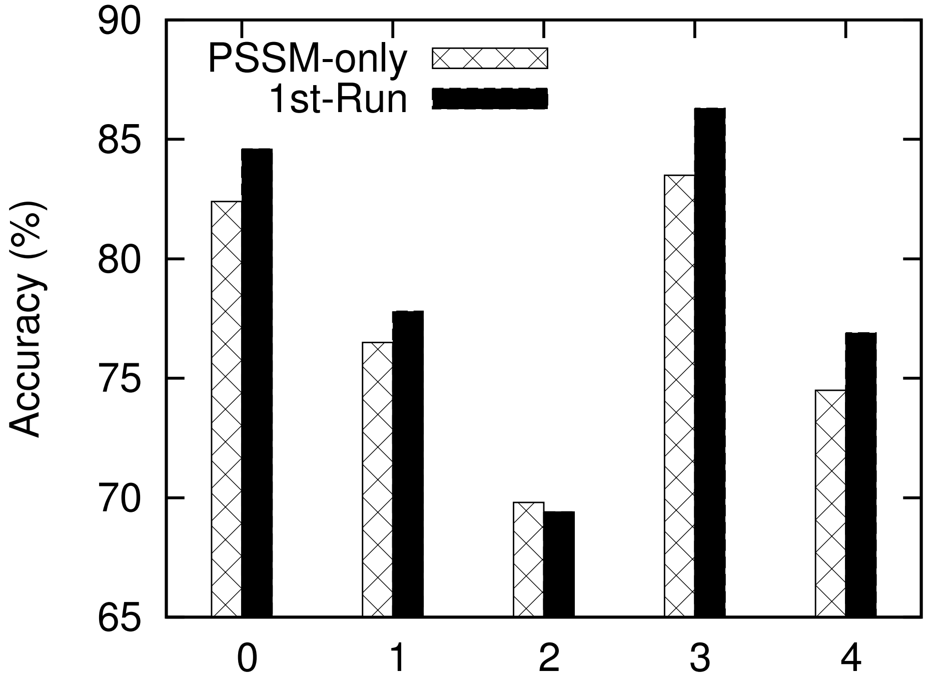

Figure 15: k-Means - Clusters: 5

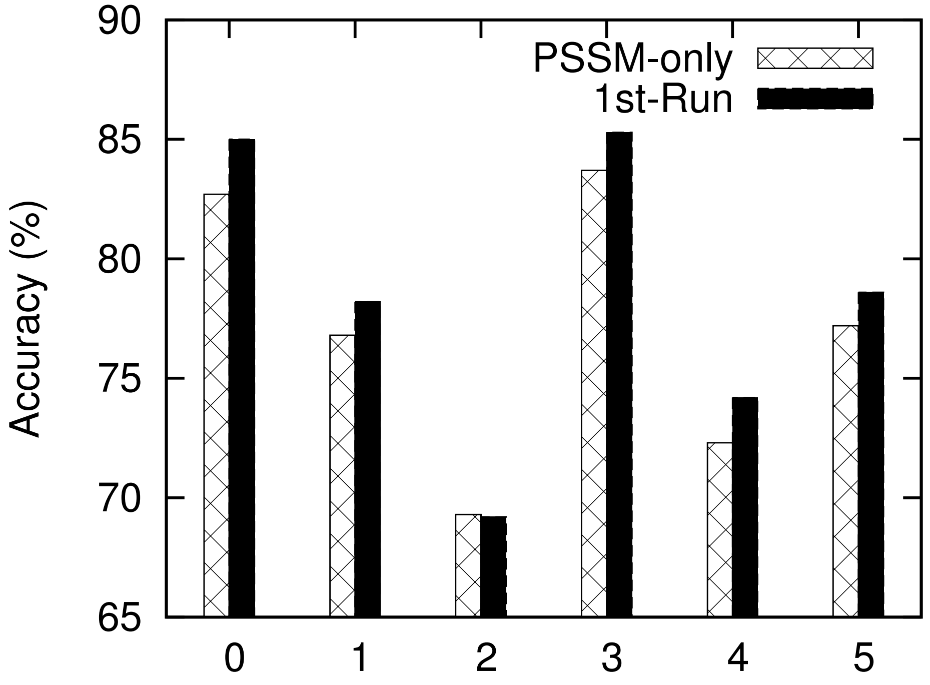

Figure 16: k-Means - Clusters: 6

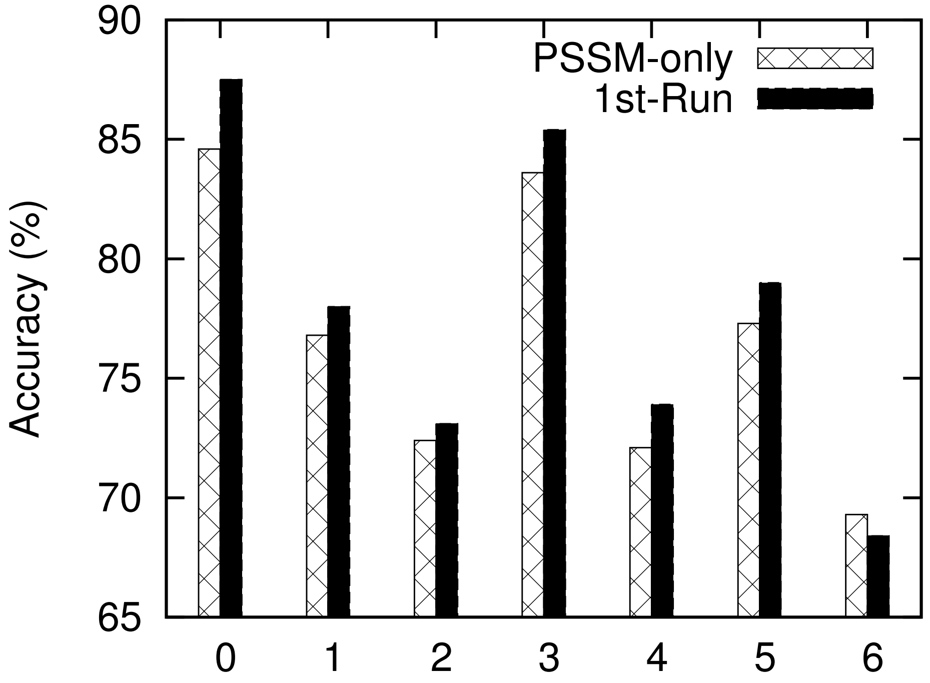

Figure 17: k-Means - Clusters: 7

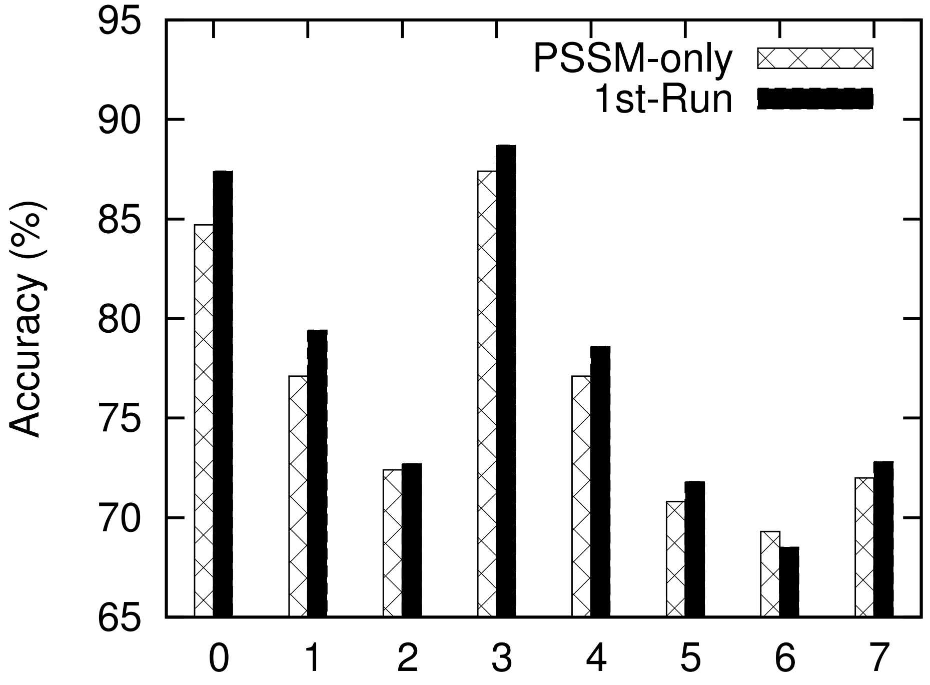

Figure 18: k-Means - Clusters: 8

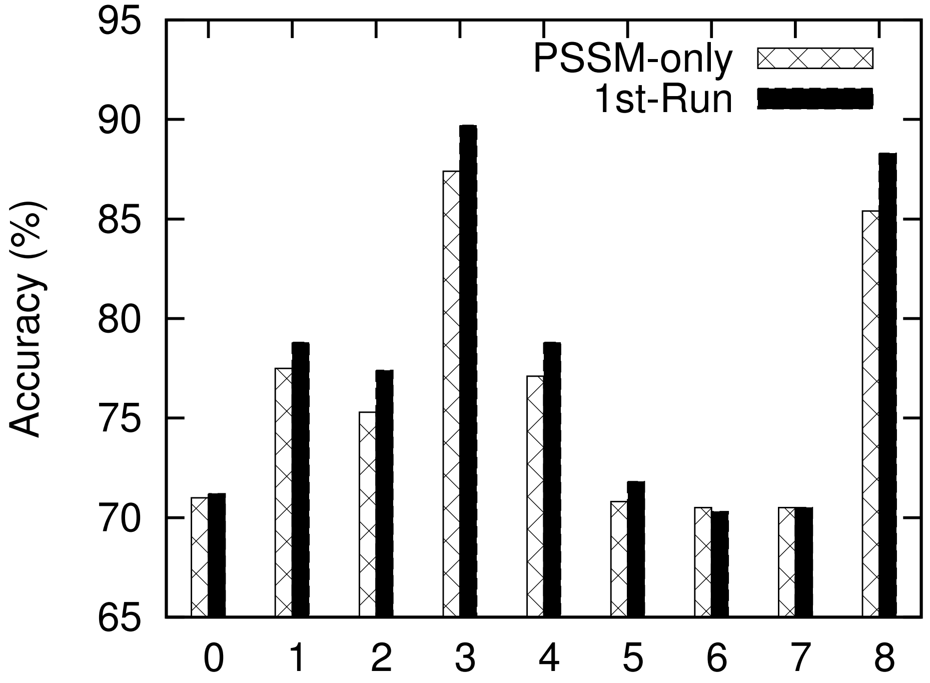

Figure 19: k-Means - Clusters: 9

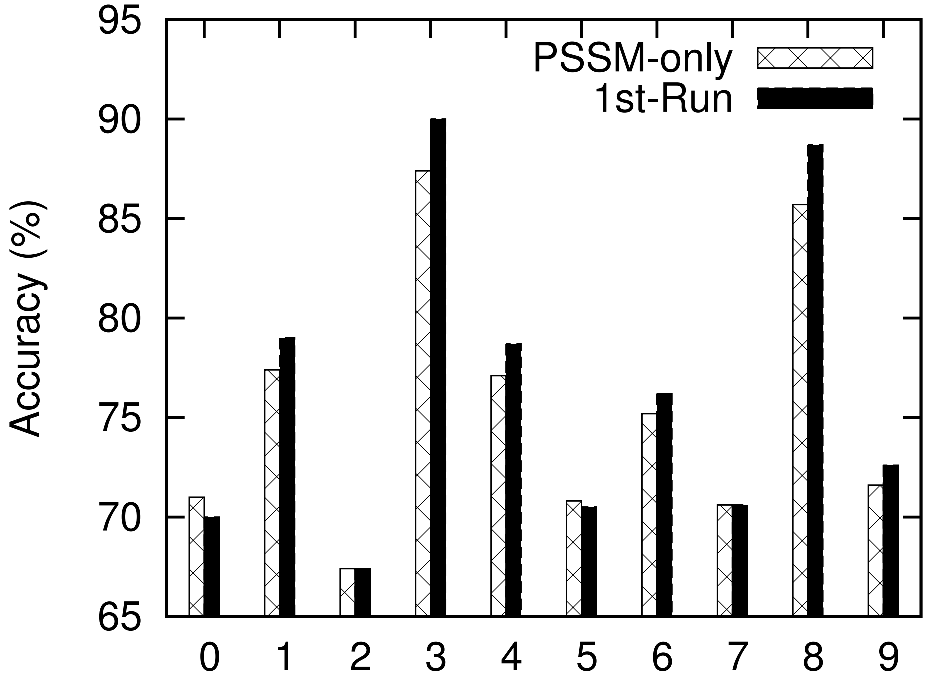

Figure 20: k-Means - Clusters: 10

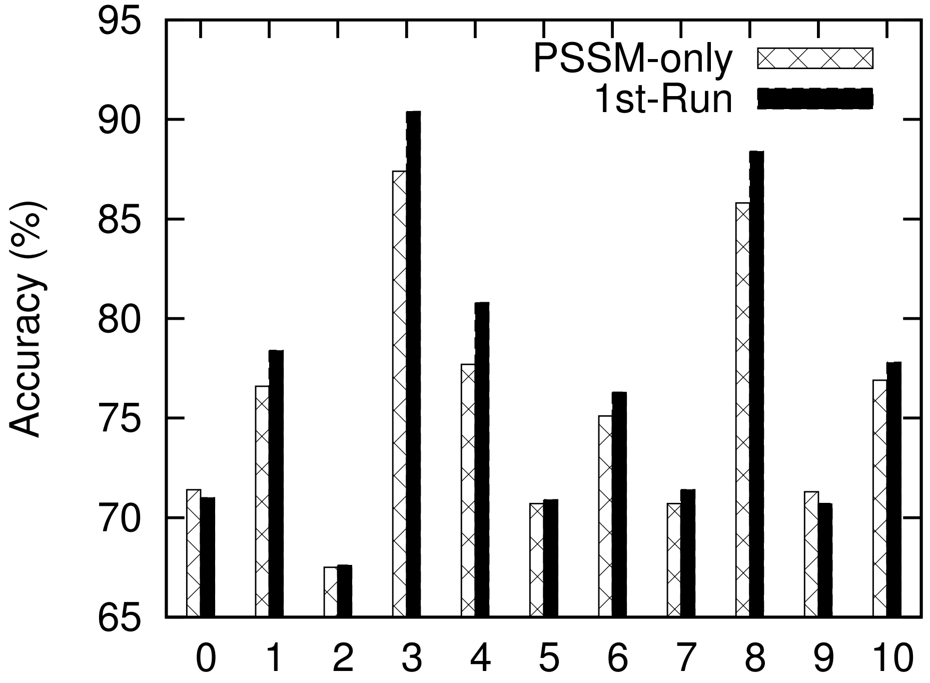

Figure 21: k-Means - Clusters: 11

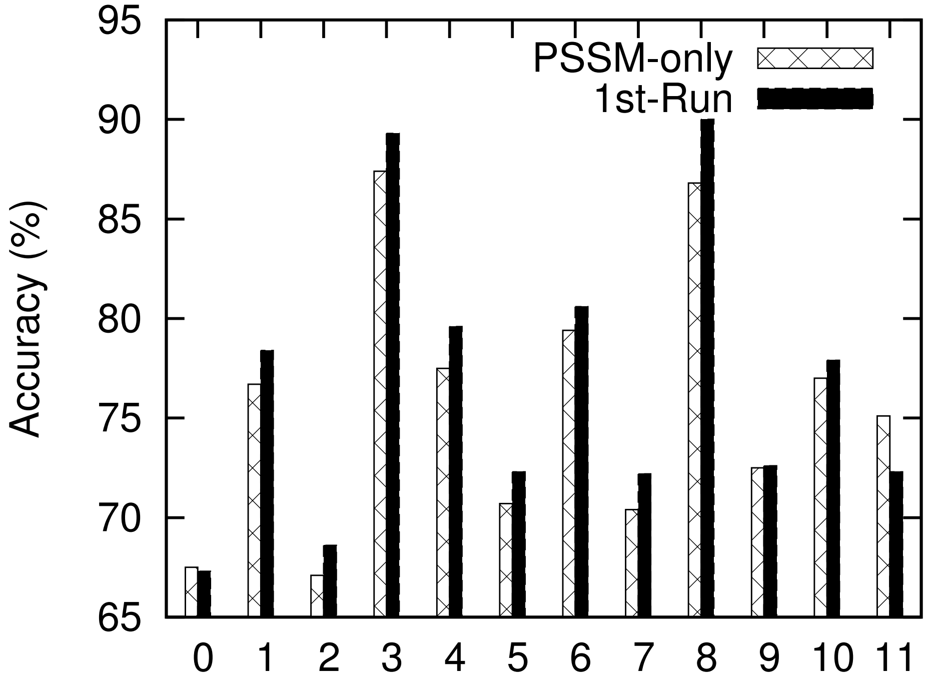

Figure 22: k-Means - Clusters: 12
