# Supplementary material for: Prediction of backbone dihedral angles and protein secondary structure using support vector machines
Source: BMC Bioinformatics. 2009 Dec 22;10:437. doi: 10.1186/1471-2105-10-437 (PMC2811710; doi:10.1186/1471-2105-10-437)
Supplement: Additional file 4 — The MAE and Q30 after the second iteration of DISSPred using EM clustering. The mean absolute errors (MAEs) and the percentage of predicted dihedral angles within 30° of the real value (Q30) for both backbone dihedral angles ϕ and ψ after two iterations of our method using EM clustering. In bold are the best results in every case. [file 1471-2105-10-437-S4.PDF]

Additional file 4

Table 1: The mean absolute error (MAE) and the percentage of predicted dihedral angles within 30° of the real value (Q_{30}) for both backbone dihedral angles ϕ and ψ after two iterations of our method using EM clustering. In bold are the best results in every case.

		Number of Clusters										
Measure	Angle	2	3	4	5	6	7	8	9	10	11	12
MAE	ϕ	29.7	27.2	27.1	26.3	25.8	**25.1**	25.7	26.0	25.6	26.1	26.5
MAE_H	ϕ	10.7	13.7	13.7	12.3	12.2	**11.3**	11.4	11.4	11.4	11.6	11.6
MAE_E	ϕ	33.3	26.9	26.4	25.0	24.1	**25.4**	25.8	26.6	26.0	26.3	27.5
MAE_C	ϕ	44.1	39.1	39.0	39.1	38.4	**36.9**	38.1	38.3	37.8	38.5	38.8
MAE	ψ	40.9	41.1	40.2	39.6	**38.5**	38.5	38.6	38.8	38.7	38.8	38.9
MAE_H	ψ	26.1	25.5	25.2	22.4	**22.3**	19.7	22.0	21.4	21.3	23.2	22.4
MAE_E	ψ	30.9	30.1	29.6	31.1	**30.7**	33.7	31.2	31.7	32.6	30.1	31.8
MAE_C	ψ	59.2	60.5	58.9	59.0	**56.7**	57.3	57.0	57.6	57.1	56.8	56.9
Q_{30}	ϕ	67.5	71.0	70.9	73.2	73.4	**75.5**	75.2	75.0	75.4	75.0	74.7
QH_{30}	ϕ	92.3	90.2	90.2	90.4	90.4	**91.9**	92.4	92.0	92.2	91.9	92.3
QE_{30}	ϕ	52.8	67.6	67.6	72.7	72.9	**72.2**	72.4	71.6	72.2	72.1	70.5
QC_{30}	ϕ	53.9	56.3	55.9	58.6	59.0	**63.3**	61.9	62.1	62.7	62.1	61.9
Q_{30}	ψ	69.3	67.8	69.8	68.9	**71.3**	70.8	70.9	70.9	71.2	70.9	71.1
QH_{30}	ψ	88.2	87.4	87.7	87.9	**88.0**	89.5	87.9	88.3	88.4	87.0	87.4
QE_{30}	ψ	78.0	78.3	78.9	77.6	**78.3**	76.2	76.9	77.5	76.9	78.2	77.9
QC_{30}	ψ	48.2	45.3	49.4	47.9	**53.0**	51.8	52.9	52.5	53.3	53.2	53.4
